# Sunburn mitigation in dragon fruit (*Hylocereus* spp.): unravelling genotype-specific physiological and biochemical responses

**DOI:** 10.3389/fpls.2025.1661147

**Published:** 2025-09-11

**Authors:** Karunakaran G., Chaturvedi Kanupriya, Manivannan Arivalagan, R. H. Laxman, Kumar Prakash, B. L. Manjunath, T. Ruchitha, K. Abhilash, Tridip Kumar Hazarika

**Affiliations:** ^1^ Division of Fruit Crops, Indian Council of Agricultural Research (ICAR)-Indian Institute of Horticultural Research, Bengaluru, Karnataka, India; ^2^ Division of Basic Sciences, Indian Council of Agricultural Research (ICAR)-Indian Institute of Horticultural Research, Bengaluru, Karnataka, India; ^3^ Division of Statistical Genetics, Indian Council of Agricultural Research (ICAR)-Indian Agricultural Statistics Research Institute (IASRI), New Delhi, India; ^4^ Department of Horticulture Aromatic and Medicinal Plants, Mizoram University, Aizawl, Mizoram, India

**Keywords:** sunburn, dragon fruit, antioxidants, Kaolin, SOD, POD, CAT, MDA

## Abstract

**Introduction:**

Sunburn is a major abiotic stress affecting dragon fruit (Hylocereus spp.), impairing tissue integrity, disrupting physiological functions, and significantly reducing yield. Developing effective mitigation strategies is critical for sustaining productivity under high radiation conditions.

**Methods:**

This study evaluated the efficacy of eleven treatments combining kaolin (5%) with shade net, seaweed extract (0.5%), and various biostimulants in red (*H. costaricensis* ‘CHESH-D1’) and white (*H. undatus* ‘CHESH-W1’) genotypes. Treatments included T1 (water spray control), T2 (kaolin 5% + green monofilament shade net 30%), and T3–T11 involving kaolin (5%) + seaweed extract (0.5%) combined individually with silica (0.5%, T4), micronutrients (0.5%, T5), petroleum oil (0.5%, T6), salicylic acid (0.5%, T7), neem soap (0.5%, T8), microbial consortium (0.5%, T10), brassinosteroids (0.5%, T11), and both neem soap (0.5%) + microbial consortium (0.5%, T9).

**Results:**

Neem soap (T8) and neem soap + microbial consortium (T9) were the most effective treatments, reducing canopy temperature by 4.2–5.1 °C and malondialdehyde (MDA) content by 32–38% compared to the control. These treatments also showed lower phenolic, flavonoid, and antioxidant enzyme (SOD, POD, CAT) activities, indicating reduced oxidative stress. Shade net + kaolin (T2) retained the highest chlorophyll content (1.82 mg g^-1 ^FW), while microbial augmentation in T9 improved nutrient uptake. The red genotype exhibited higher tolerance to sunburn, with 12% lower MDA levels and 18% higher SOD activity than the white genotype.

**Discussion:**

Kaolin-based treatments, particularly when combined with neem soap and microbial amendments, synergistically alleviated sunburn stress by reducing oxidative damage, improving antioxidant enzyme activity, and enhancing nutrient homeostasis. Genotype-specific responses highlight the potential for precision management strategies to improve dragon fruit resilience under high radiation environments.

## 1 Introduction

Dragon fruit (*Hylocereus* spp.), a member of the Cactaceae family, is rapidly gaining global prominence due to its exceptional nutritional value, economic potential, and ecological adaptability. Native to the tropical and subtropical rainforests of southern Mexico, Guatemala, and Costa Rica (Mizrahi et al., 1997), its cultivation has expanded significantly across Asia (Vietnam, China, Malaysia, Taiwan), the Middle East (Israel), and Australia. In Latin America, Colombia and Ecuador, particularly the latter for its premium yellow-skinned varieties, are emerging as major producers (Mitra and Pathak, 2024). Rich in bioactive compounds such as vitamins, potassium, betacyanin, and phenolic acids (Arivalagan et al., 2021), the fruit holds promise in combating diabetes, dyslipidemia, metabolic syndrome, cardiovascular diseases, and cancer. Furthermore, its uses extend to the food and nutraceutical industries, including natural colorants, eco-friendly packaging, edible films, photoprotective products, and functional additives (Nishikito et al., 2023).

Among the commonly cultivated species: *Hylocereus undatus* (white pulp), *H. costaricensis* (red pulp), and *H. megalanthus* (yellow skin) distinct physiological adaptations are evident. *H. megalanthus* thrives under higher shade intensities (50–60%), while *H. costaricensis* performs better in lower shade conditions (30%) (Patil et al., 2024). These differences are due to structural traits: *H. megalanthus* lacks a waxy layer and sunken stomata, making it sensitive to high light intensity, whereas *H. costaricensis* possesses a thicker wax layer and sunken stomata, enhancing its tolerance to intense sunlight and heat (Yadav et al., 2025).

India has witnessed rapid expansion in dragon fruit cultivation in recent years, with over 5,000 hectares now under production across major states including Karnataka, Maharashtra, Gujarat, West Bengal, Andhra Pradesh, Telangana, and parts of North and Northeast India (Karunakaran et al., 2019). Despite this growth, domestic production still falls short of rising demand, largely due to the crop’s vulnerability to abiotic stresses, particularly sunburn under high-temperature conditions typical of tropical, semi-arid, and arid regions. Unlike true desert cacti, dragon fruit evolved in the shaded understories of tropical rainforests and is thus more sensitive to heat stress (Munné-Bosch and Vincent, 2019). The optimal temperature for *H. undatus* (30°C day/20°C night) (Chu and Chang, 2020) is frequently exceeded during peak summer (March to June), when temperatures in major production zones often surpass 38°C. Dragon fruit’s most critical growth phases coincide with this high-stress period, increasing its susceptibility to sunburn—an issue also reported in other fruit crops like apples (Racsko and Schrader, 2012; Gambetta et al., 2021), grapes (Krasnow et al., 2010), and pomegranates (Melgarejo-Sánchez et al., 2015; Al-Saif et al., 2022). In dragon fruit, sunburn symptoms are primarily observed on cladodes such as cladode chlorosis, bleaching, necrosis, poor flower bud initiation, and reduced fruit set impairing growth and productivity (Nobel and De la Barrera, 2002; Chang et al., 2016; Chien and Chang, 2019; Kakade et al., 2023). Climate change is expected to exacerbate these challenges, with increasing temperature variability and drought frequency projected to heighten sunburn risk and threaten long-term crop sustainability and farmer livelihoods (Ayangbenro and Babalola, 2021).

As a CAM plant, dragon fruit conserves water by closing stomata during the day, which limits transpirational cooling and causes internal heat accumulation. This compromises photosynthetic efficiency (Chang et al., 2016; Nobel and De la Barrera, 2002), reduces reproductive potential (Nerd et al., 2002), and increases susceptibility to pathogen infection (Chien and Chang, 2019). Sunburn-induced tissue damage is often a result of photo-oxidative stress caused by ROS accumulation, manifesting as yellowing, browning, or necrosis (Dawood and Latef, 2023). High temperatures disrupt chloroplast structures, degrade thylakoid membranes, and increase membrane permeability, impairing photosynthesis and accelerating senescence (Kakade et al., 2023). These effects are further amplified by decline in chlorophyll concentration and increase in photoprotective pigments (e.g., xanthophylls), which alter tissue coloration. The resulting oxidative stress overwhelms cellular defenses (Bita and Gerats, 2013), despite native antioxidant responses such as redox adjustments and phenolic induction (Lal and Sahu, 2017). Prolonged heat stress impairs chlorophyll biosynthesis (Nuzhyna et al., 2018; Soengas et al., 2018), triggers membrane lipid peroxidation (measured via MDA), and alters antioxidant enzyme activity (Jbir-Koubaa et al., 2015). Plants counteract these effects through enzymatic and non-enzymatic antioxidants, including flavonols, carotenoids, and anthocyanins, which scavenge ROS and protect chloroplasts (Solovchenko et al., 2010). Therefore, quantifying these biochemical markers provides crucial insight into stress tolerance and adaptation.

With the projected rise in global temperatures and frequency of heat waves, the adoption of cost-effective, climate-resilient agricultural strategies has become essential. While techniques like shade nets (Patil et al., 2024; Afonso et al., 2025) and sprinkler irrigation (Do et al., 2024) offer relief from heat stress, their use is often constrained by high water demand, costs, and disease risks (Manja and Aoun, 2019). As a result, alternative interventions such as improved canopy management (Boini et al., 2025), trellis design (Danko et al., 2024), and optimized row orientation (Amogi et al., 2025) are gaining importance. Recent studies specific to dragon fruit support these approaches. Patil et al. (2024) showed that shade nets improved yield and fruit quality under semi-arid conditions, while Doke et al. (2024) demonstrated that pruning enhanced light distribution, reduced sunburn, and limited disease pressure. Fischer et al. (2022) further emphasized the global rise in sunburn-related disorders in tropical fruits, highlighting the need for integrative solutions. Agroforestry-based systems, where dragon fruit is co-cultivated under trees like mango (*Mangifera indica*) or aonla (*Emblica officinalis*), have also shown promise in moderating microclimates and improving sustainability (Kishore et al., 2025; Reza et al., 2022). Kaolin-based particle films offer another innovative solution. Kaolin (Al_2_Si_2_O_5_(OH)_4_) creates a reflective barrier that reduces UV and infrared radiation while allowing photosynthetically active radiation to pass through, without impairing gas exchange (Brito et al., 2019; Wang et al., 2022). Its application has been shown to reduce tissue temperature, maintain chlorophyll content, and promote plant growth under heat stress (Mahmoudian et al., 2021; Teker, 2023; Santos and Coelho Filho, 2024).

Beyond sunburn, compromised cuticle integrity increases susceptibility to fungal infections and physiological decline (Contreras et al., 2008; Fischer et al., 2022; Manja and Aoun, 2019). To simultaneously address abiotic and biotic stress, this study integrates kaolin sprays with biological and organic amendments. Seaweed extract was included for its known role in enhancing antioxidant systems, osmotic balance, and enzymatic defenses under abiotic stress (Ali et al., 2021; Kumar et al., 2024), while neem soap offered broad-spectrum antifungal and insecticidal properties with low environmental toxicity (Wylie and Merrell, 2022). A microbial consortium composed of plant growth-promoting rhizobacteria was also applied to enhance nutrient acquisition, root health, and systemic immunity (Santoyo et al., 2021; Cruz et al., 2025). These treatments were hypothesized to act synergistically by reducing heat load, mitigating ROS-induced damage, and improving plant resilience through enhanced physiological and microbial defence pathways.

Despite the growing interest in dragon fruit and its increasing cultivation across climatic zones, no previous studies have systematically evaluated sunburn mitigation strategies using a combination of kaolin-based sprays and biostimulants. Furthermore, genotype-specific physiological responses to heat stress remain underexplored. Existing studies focus primarily on shading or irrigation effects but lack insights into how foliar treatments with biocompatible agents like seaweed, neem, PGPR, and natural oils influence antioxidant defence and nutrient balance.

To bridge these knowledge gaps, this study presents the first comprehensive field evaluation of kaolin-based treatments—alone and in combination with organic biostimulants—for mitigating sunburn in red (*H. costaricensis* ‘CHESH-D1’) and white (*H.undatus* ‘CHESH-W1’) dragon fruit genotypes. By assessing physiological parameters (canopy temperature, chlorophyll retention) and biochemical responses (MDA, antioxidant enzymes, phenolic and flavonoid content), we aim to identify cost-effective and climate-resilient strategies that support sustainable dragon fruit production in sunburn-prone environments.

## 2 Material and methods

### 2.1 Experimental site and weather conditions

The study was conducted over two consecutive years (2023–2024) in a dragon fruit block at the Indian Institute of Horticultural Research (IIHR), Bengaluru, Karnataka, India (13.15°N, 77.49°E; elevation: 890 m above sea level). The region experiences a subtropical climate, with monthly temperatures ranging from 26°C (December minimum) to 36°C (April maximum) (**Supplementary Table 1**). The mean temperature recorded during the study period was 24.92°C. Relative humidity fluctuated seasonally, reaching 90% in July (peak monsoon) and dropping to 32% in March (driest month). Average wind speed was 3.94 km h^-^¹, occasionally peaking at 1.5 m s^-1^ while daily sunshine duration extended to 12–14 hours during summer. The experimental site featured sandy loam soil with slightly acidic pH (~5.6) and organic carbon content of ~0.9%. Weather data for this study were recorded at the Climatological Station located within ICAR-IIHR, ensuring site-specific accuracy in meteorological observations.

### 2.2 Plant material and treatments

The study utilized three-year-old plants of two dragon fruit genotypes developed by ICAR-IIHR, Central Horticultural Experiment Station, Hirehalli (**Supplementary Figure 1**): (i) CHESH-W1 (*Hylocereus undatus*), a photosensitive white-pulp selection producing oval fruits (450–550 g) with TSS of 11–12°Brix and a yield potential of 42.5 kg/pole; and (ii) CHESH-D1 (*H. costaricensis*), a photo-insensitive red-pulp selection yielding round fruits (350–400 g) with higher TSS (14.5°Brix) and productivity of 49.5 kg/pole (Karunakaran et al., 2024). Prior to planting, the soil was amended with 10 kg of farmyard manure, 250 g of neem cake, and 250 g of single super phosphate per pole. After establishment, lateral growths were pruned to promote vertical climbing on support structures. Plants were trained using a single reinforced concrete pole system with cement rings, spaced uniformly at 3 × 3 m (1110 poles/ha), accommodating four plants per structural unit (**Supplementary Figure 2**). Eleven treatments (T1–T11) were evaluated for sunburn mitigation, comprising combinations of reflectants, physical barriers, neem-oil, bio-stimulants, and microbial agents as presented in table form. The Arka Microbial Consortium, developed by ICAR-IIHR, contained *Pseudomonas fluorescens*, *Bacillus subtilis*, *Azospirillum brasilense*, and *Trichoderma harzianum* (1:1:1:1 ratio) applied at 10^6^–10^7^ CFU/mL. All sprays were applied during early morning to minimize phytotoxicity risks. Details of treatments used for evaluation of sunburn mitigation in dragon fruit:

T1. Water spray (control)
T2. Kaolin (5%) + Green shade net (30%)
T3. Kaolin (5%) + Seaweed (0.5%)
T4. Kaolin (5%) + Seaweed (0.5%) + Silica (0.5%)
T5. Kaolin (5%) + Seaweed (0.5%) + Micronutrients (0.5%)
T6. Kaolin (5%) + Seaweed (0.5%) + Petroleum oil (0.5%)
T7. Kaolin (5%) + Seaweed (0.5%) + Salicylic acid (0.5%)
T8. Kaolin (5%) + Seaweed (0.5%) + Arka Neem soap (0.5%)
T9. Kaolin (5%) + Seaweed (0.5%) + Arka Neem soap (0.5%) + Arka Microbial Consortium (0.5%)
T10. Kaolin (5%) + Seaweed (0.5%) + Arka Microbial Consortium (0.5%)
T11. Kaolin (5%) + Seaweed (0.5%) + Brassinosteroids (0.5%)

### 2.3 Canopy temperature and light interception

The canopy temperature observations were recorded by using VarioCAM^®^ HD head 600 infrared camera through thermal imaging in different treatments. The camera features a high-resolution uncooled microbolometer focal plane array, operating within the 7.5 to 14 µm spectral range. It provides dependable thermal readings, while its thermal resolution of 0.03 K at 30°C allows precise detection of minor temperature fluctuations. The thermal images were obtained from canopy top during 12 noon to 12.30 pm. The average canopy temperature was extracted using IRBIS software. The light interception under shade net and outside shade net was determined on clear days by measuring photosynthetically active radiation (PAR) at IST 13:00 hours, utilizing a Li-Cor Li-190SA quantum sensor instrument (Li-Cor, Lincoln, NE) attached to a data receiver, following the method outlined by Flenet et al. (1996).

### 2.4 Quantification of chlorophyll pigments

Chlorophyll a, b, and total chlorophyll content in cladode tissue were estimated using dimethyl sulfoxide (DMSO) following the protocol of Inskeep and Bloom (1985), with modifications for succulent tissue. Fresh samples (1 g) were incubated in 7.0 mL DMSO at 65°C for 60 minutes. The supernatant was adjusted to 10 mL with DMSO, and absorbance measured at 480, 645, and 663 nm (DMSO blank). Concentrations (mg g^-^¹ FW) were calculated as:


$$\text{Chlorophyll a}=\left[\left(12.7\times {\text{A}}_{663}\right)-\left(2.69\times {\text{A}}_{645}\right)\right]\times \left(\text{V}/\text{W}\right)$$

$$\text{Chlorophyll b}=\left[\left(22.9\times {\text{A}}_{645}\right)-\left(4.68\times {\text{A}}_{663}\right)\right]\times \left(\text{V}/\text{W}\right)$$

Total chlorophyll=Chlorophyll a+Chlorophyll b

where V=final volume (10 mL) and W=sample weight (1 g).

### 2.5 Percent sunburn and disease incidence

Sunburn and disease incidence were evaluated *in situ* using standardized visual protocols adapted from Chang et al. (2016). Sunburn severity was characterized by chlorotic-necrotic lesions on sun-exposed cladodes, with incidence calculated as the percentage of affected cladodes per plant. Sunburn was quantified as:


$$\begin{array}{c} \text{Sunburn }\left(\%\right)=(\text{Number of cladodes with sunburn symptoms}/\\ \text{Total observed cladodes})\times 100 \end{array}$$

Disease Severity: To evaluate pathogen susceptibility, Percent Disease Index (PDI) was calculated for stem canker (*Neoscytalidium dimidiatum*) and stem rot (*Fusarium* spp.). Symptoms were assessed using a standardized 0–5 scale based on lesion progression and necrosis extent:

0. No visible symptoms
1. 1–10% area infected (minor localized lesions)
2. 11–25% area infected (small sunken lesions)
3. 26–50% area infected (moderate necrosis)
4. 51–75% area infected (large necrotic regions)
5. >75% area infected (severe necrosis/rot)

The PDI for each species and disease type was calculated using the following formula:


$$\begin{array}{c} \text{PDI}=[\sum \left(\text{Disease rating × Number of plants in that rating}\right)÷\\ \left(\text{Total number of plants observed}\times \text{ Maximum disease rating})\right]\\ \times 100 \end{array}$$

### 2.6 Estimation of phenols, flavonoids, antioxidants, activity of antioxidant enzymes and lipid peroxidation

For phytochemical analysis, approximately 5 g of fresh dragon fruit tissue from each genotype was extracted with 10 mL of 80% aqueous ethanol (v/v) using ultrasonic-assisted extraction (Labocon LUC104) at 60°C for 30 min in the dark. After centrifugation at 5,000 × g for 15 min, the supernatant was collected and the residue was re-extracted twice. The combined supernatants were concentrated using a rotary evaporator (Heidolph Hei-VAP Advantage) and redissolved in 5 mL of distilled water before storage at -20°C until analysis. Total phenolic content was determined using the Folin-Ciocalteu method (Singleton et al., 1999), with results expressed as mg gallic acid equivalents (GAE) per 100 g fresh weight. Total flavonoid content was measured according to the aluminium chloride colorimetric assay (Zhishen et al., 1999). Antioxidant capacity was evaluated through three complementary assays: DPPH radical scavenging activity was assessed following Brand-Williams et al. (1995), with results calculated as: % scavenging [(Acontrol-Asample)/Acontrol] × 100 and expressed as SC50 values (μmol Trolox equivalents/100 g FW); FRAP assay was performed according to Benzie and Strain (1996) by measuring absorbance at 593 nm; and CUPRAC activity was determined following Apak et al. (2004) with absorbance measurements at 450 nm. Data from FRAP assay is shown in results; other assays showed similar trends. Antioxidant enzyme activities, including superoxide dismutase (SOD), peroxidase (POD), and catalase (CAT), were assayed according to Zhang and Kirkham (1996). The assay was performed using potassium phosphate buffer (pH 7.8), prepared by mixing 90 mL of Solution A (1.22 g KOH in 100 mL water) with 10 mL of Solution B (2.422 g KH_2_PO_4_ in 100 mL water) and adjusting to a final volume of 200 mL. Reaction mixtures contained 1.6 mL buffer, 0.1 mL Na_2_CO_3_ (1% w/v), 0.3 mL NBT (0.1% w/v), 0.3 mL methionine (0.1% w/v), 0.1 mL enzyme extract, 0.3 mL EDTA (0.1% w/v), and 0.3 mL riboflavin (0.1% w/v). Tubes were covered with aluminum foil to prevent light interference, and absorbance was measured at 560 nm. The reaction mixture consisted of 2.55 mL phosphate buffer (pH 7.0), 0.2 mL ortho-phenylenediamine (OPD; 0.05% w/v), 0.2 mL H_2_O_2_ (0.1% v/v), and 0.05 mL enzyme extract, adjusted to a final volume of 3 mL. Absorbance was recorded at 290 nm. CAT activity was measured following the POD protocol but without OPD, and absorbance was read at 310 nm. Lipid peroxidation was assessed by quantifying MDA content using the thiobarbituric acid (TBA) method (Heath and Packer, 2022). Briefly, 1 mL of supernatant was mixed with 3 mL of 20% trichloroacetic acid containing 0.5% TBA, heated at 95°C for 30 min, and cooled on ice. After centrifugation (10,000 × *g*, 10 min), absorbance was measured at 532 nm, subtracting nonspecific absorption at 600 nm. MDA concentration was calculated using an extinction coefficient of 155 mM^-^¹ cm^-^¹.

### 2.7 Estimation of mineral content

Samples of control and T8, T9 treated plants were processed, separated and dried in the oven at 60°C to constant weight procedure as described by Piper (1966), grinded in porcelain pestle and mortar and stored in air tight containers. The analysis was carried out using three independent replications for each accession. The concentration of nitrogen in samples was determined by Kjeldhal’s method (KjeltekAut-Analyzer, Gerhardt, Germany), phosphorous by Vanadomolybdate method using UV-visible Spectrophotometer (Shimadzu UV-1900i, Milton Keynes MK12 SRE, UK) and potassium by flame photometer. The concentration of calcium, magnesium and micronutrients were determined using Atomic Absorption Spectrophotometer (AAS 280 FS Agilent Technologies, Santa Clara, USA) by wet digest method with HNO3 and HCLO4 in 10:4 ratio.

### 2.8 Microscopic analysis of stomatal and epicuticular wax structures

Mature cladodes of control and treated plants were collected from all directions of the dragon fruit plant at 11:00 a.m. (IST). The sampled cladodes were sectioned into small pieces (1 cm² surface area, 1 mm depth) and mounted on copper tape for microscopic analysis. Stomatal and epicuticular wax structures were examined using two imaging systems: iMOS Microscope: Coupled with a MiaCam camera, this system was used to document changes in stomatal morphology across treatments at varying magnifications (e.g., ×200, ×500). TM3030Plus Tabletop Scanning Electron Microscope (SEM, Hitachi, Japan): High-resolution SEM imaging was performed to analyze fine structural details of both stomata and epicuticular wax deposits.

### 2.9 Statistical analysis

The experiment was laid out in completely randomized block design with eleven treatments and replicated thrice with twelve poles per replication. The data was collected from three technical replicates of each genotype for all parameters which was subjected to analysis of variance (ANOVA) and significant differences were evaluated at 5% level of significance followed by least significant difference test in ‘R’ studio for Windows (Versions 4.1.1 and 1.4.1417). Principal component analysis (PCA), cluster heat map and correlation analyses (Pearson test) were performed using ‘ggplot2’ library.

## 3 Results

### 3.1 Effect of treatments on the canopy temperature, chlorophyll content, sunburn and disease incidence

**Figure 1:**
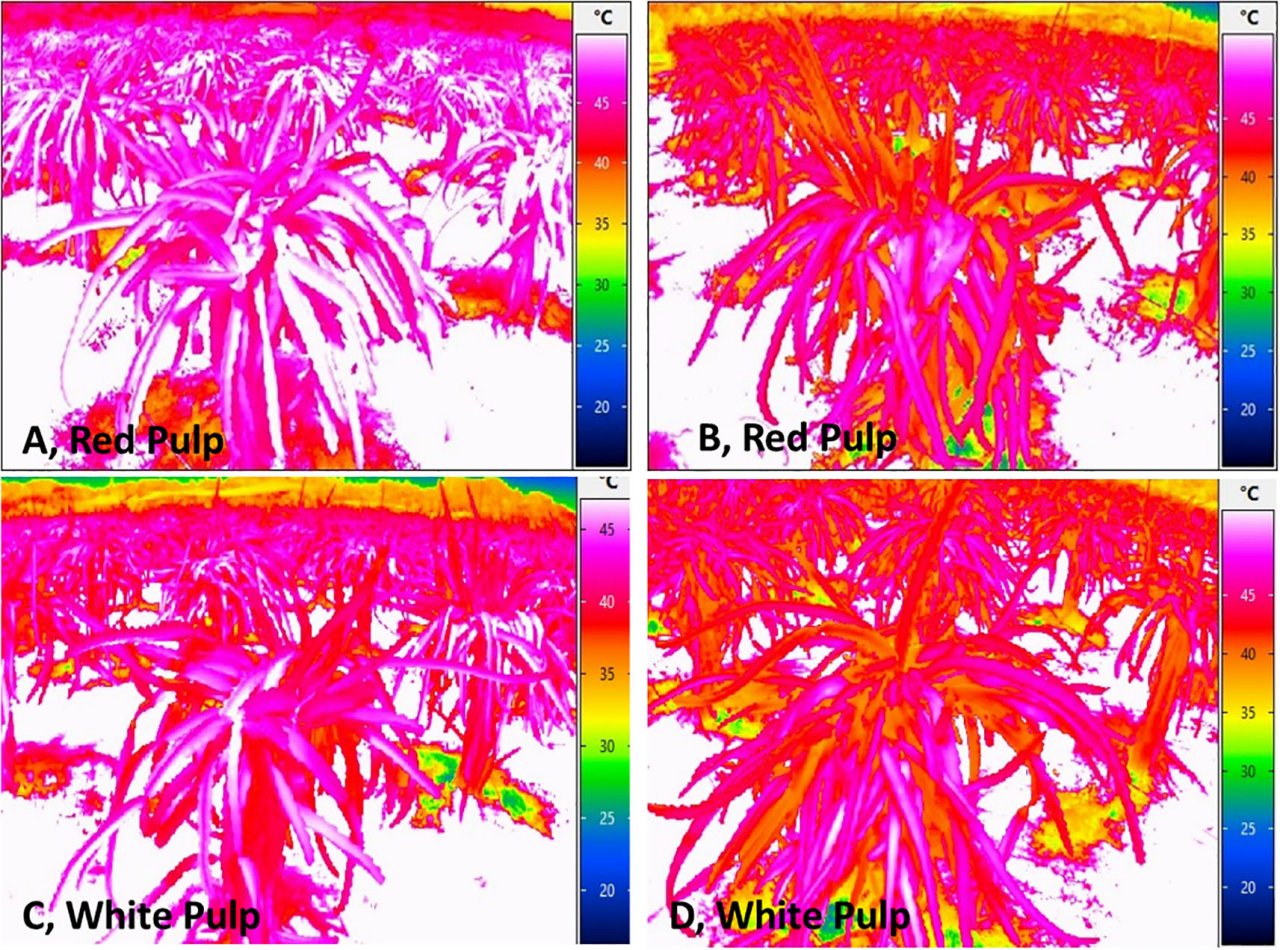
Thermal imaging of red and white pulp dragon fruit canopy temperature under different treatments. **(A, C)** T1: Water spray (control) showed higher canopy temperatures, with most cladodes exceeding 40°C **(B, D)** T8: Kaolin (5%) + Seaweed (0.5%) + Neem soap (0.5%) treatment demonstrated lower canopy temperatures, suggesting improved heat mitigation and reduced thermal stress. The color gradient represents temperature variations, with warmer tones (pink/red) indicating higher temperatures and cooler tones (blue/green) indicating lower temperatures.

Protective spray treatments revealed significant differential responses (*P< 0.01*) across all measured parameters viz., canopy temperature, chlorophyll content, sunburn incidence, and disease resistance. Thermal imaging of the plants was utilized to record the canopy temperatures under stress. The control condition (T1) resulted in the highest canopy temperatures in both genotypes, with 43.73°C in the white pulp genotype and 41.30°C in the red, indicating greater heat stress under untreated conditions (**Table 1**, **Figure 1**). In contrast, T8 (kaolin + seaweed + neem soap 0.5%) significantly reduced canopy temperatures by 12.2% in the white pulp genotype (38.40°C) and by 12.6% in the red pulp genotype (36.08°C), the lowest among all treatments. Kaoline + seaweed-based neem soap was found to be most effective in temperature regulation, with the red pulp genotype exhibiting inherently better thermal tolerance across treatments.

**Table 1:** Effect of spray treatments on the canopy temperature and chlorophyll content of white and red pulp dragon fruit.

Treatment	Canopy temp		Chlorophyll a		Chlorophyll b		Total chlorophyll	
	(°C)		(mg g-1 FW)		(mg g-1 FW)		(mg g-1 FW)	
	White	Red	White	Red	White	Red	White	Red
T1	43.73a	41.30b	1.42h	2.02f	0.32i	0.32i	1.74j	2.34ghi
T2	39.50b-g	38.02gh	2.13cd	2.21bc	0.51bc	0.40fg	2.64bc	2.72cd
T3	40.26b-e	40.17b-f	1.71f	2.01f	0.42f	0.41g	2.13i	2.43fg
T4	40.72bc	39.51b-g	1.43h	1.97f	0.40f	0.41fg	1.83j	2.37fgh
T5	40.44b-d	39.15c-g	1.92f	2.03def	0.50d	0.30h	2.42fg	2.53ef
T6	40.42b-d	39.31b-g	1.73g	1.96f	0.41e	0.32i	2.14hi	2.37fgh
T7	39.84b-g	38.24fg	2.03f	2.15cde	0.53cd	0.41fg	2.56ef	2.68de
T8	38.40e-g	36.08h	2.41a	2.31ab	0.81a	0.40e	3.22a	2.82b
T9	39.34b-g	38.00gh	2.22bc	2.31abc	0.51b	0.33fg	2.73b	2.82bc
T10	41.00bc	40.10b-f	1.51h	2.03def	0.32i	0.40fg	1.83j	2.35ef
T11	40.03b-f	38.46d-g	2.04ef	2.12cd	0.50cd	0.42fg	2.54de	2.62cd

Values with the same upper case were not significantly different in LSD test *(p< 0.05).*

The contents of chlorophyll ‘a’ (Chl-a), chlorophyll ‘b’ (Chl-b) and total chlorophyll (Chl) were assessed to understand the behavior of photosynthetic pigments under heat and light stress. The control (T1) recorded the lowest Chl-a content (1.4 mg g^-1^FW in white and 2.0 mg g^-1^FW in red), while T8 significantly increased Chl-a by 71.4% in the white pulp genotype (2.4 mg g^-1^FW). Notably, the red pulp genotype consistently maintained higher Chl-a across treatments, suggesting superior photosynthetic stability. Chl-b followed a different trend, with the white pulp genotype recording higher levels than red in most treatments. The highest total chlorophyll content was observed under T8 in the white pulp genotype (3.3 mg g^-1^FW), reflecting an 83.3% increase over the control, followed by T9 (55.6% increase) and T2 (55.6% increase) (**Table 1**).

**Figure 2:**
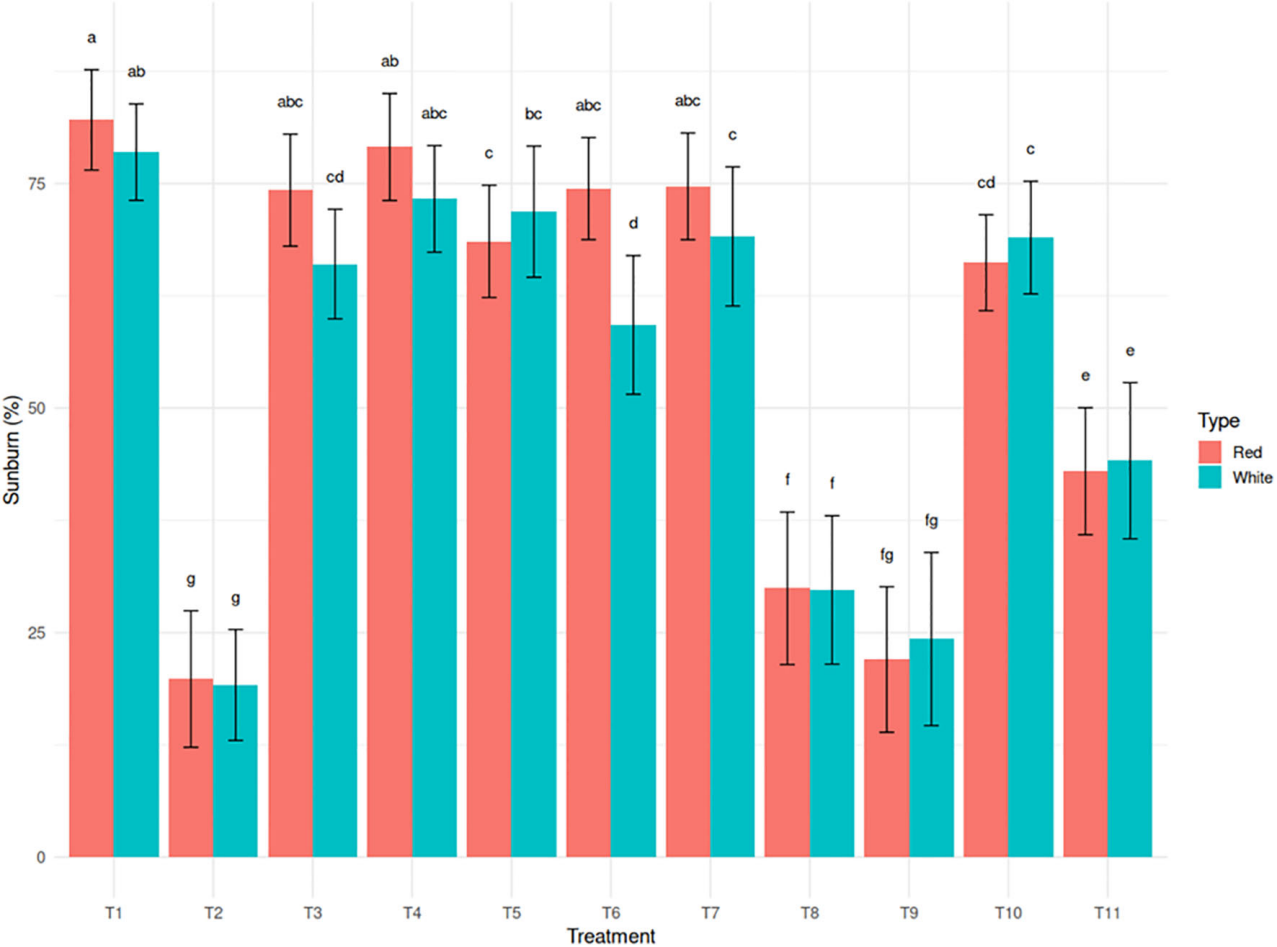
Effect of sunburn mitigation treatments (T2-T11) on sunburn injury (%) in red and white dragon fruit genotypes compared to untreated T1 (control).

Excessive exposure to sunlight, combined with high temperatures, resulted in visible tissue damage on cladodes, often manifesting as yellowing. Light intensity was 1348.05 µmol photons m^-^² s^-^¹ in the open field and 633.76 µmol photons m^-^² s^-^¹ under the shade net, representing a 53% reduction in photosynthetically active radiation under a 30% green shade net. Sunburn incidence decreased significantly under shade net and protective spray treatments. The untreated control recorded the highest sunburn incidence (78.6% in white and 82.1% in red). In contrast, T2 (Kaolin + Green Shade Net) exhibited the greatest reduction, limiting sunburn to 19.3% in white and 19.8% in red (75.9% reduction) (**Figure 2**). Kaolin based neem treatments such as T8 and T9 also showed significant reductions in sunburn incidence (61.8% reduction for T8 in white and 73.2% reduction for T9 in red). These trends were strongly correlated with reduced canopy temperature and improved chlorophyll retention, underscoring the physiological importance of these parameters in sunburn mitigation.

**Figure 3:**
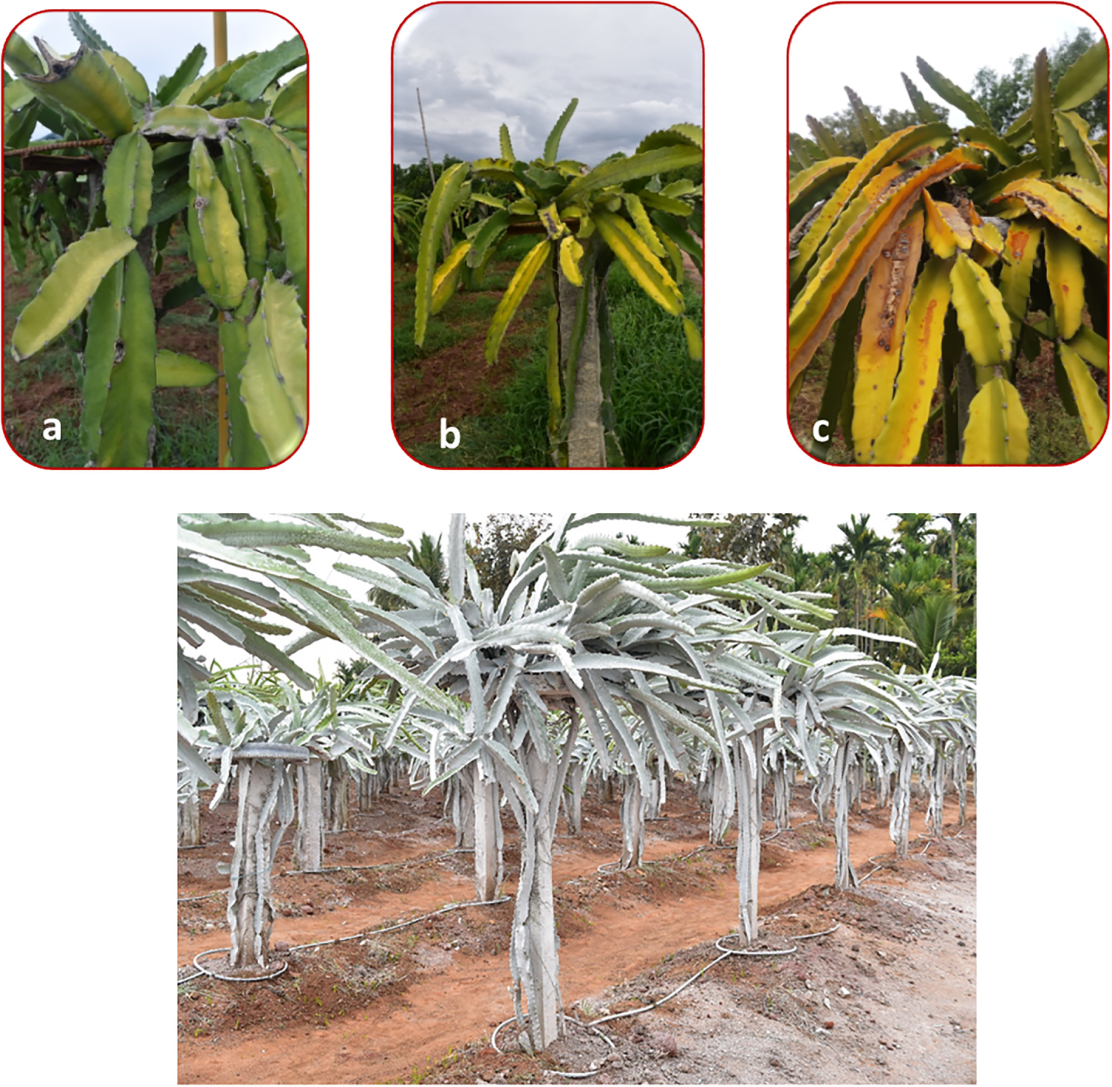
Top panel showing the progress of **(a)** yellowing, **(b)** sunburn and **(c)** stem rot in untreated plants. Bottom picture shows the treated plants free from sunburn and disease symptoms.

**Figure 4:**
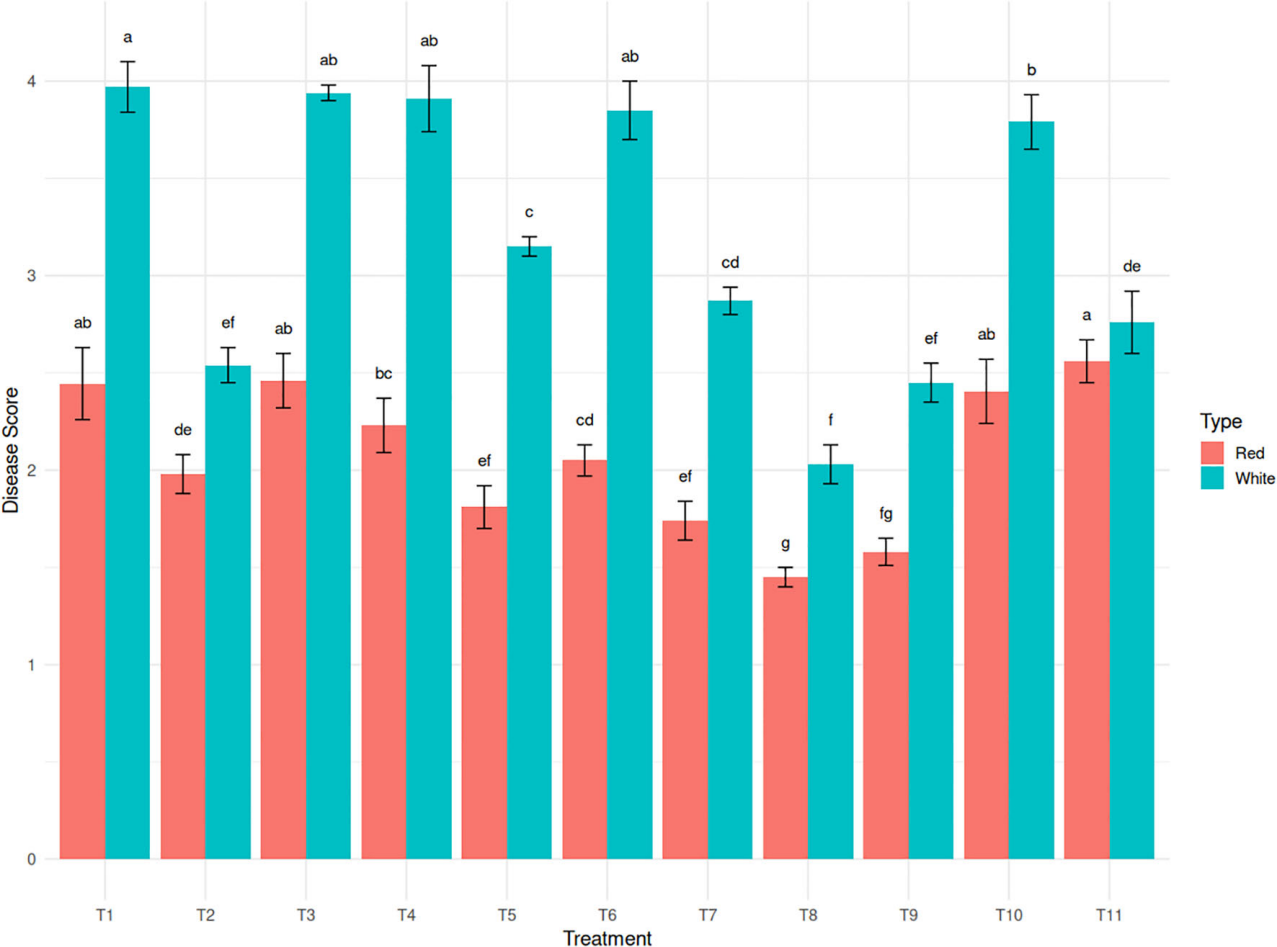
Effect of sunburn mitigation treatments (T2-T11) on disease incidence in red and white dragon fruit genotypes compared to untreated T1 (control).

Sunburn is known to increase the incidence of diseases in affected plants. Sunburn weakens plant tissues, making them more susceptible to secondary infections by fungi, bacteria, and other pathogens (**Figure 3**). Disease incidence followed similar trends as sunburn injury, with the white pulp genotype exhibiting a significant 49.6% reduction under T8 (2.03) and 37.0% reduction under T2 (2.54), compared to a high disease score of 4.03 in the control (**Figure 4**). Interestingly, while neem soap proved most effective in the white pulp genotype, the red pulp genotype responded better to T11 (Kaolin + Seaweed + Brassinoids), recording a disease score of 2.56. This suggests a genotype-specific hormonal response potentially mediated by brassinosteroids.

Among the two genotypes, the red pulp genotype displayed better inherent tolerance across most parameters, including consistently lower canopy temperatures, higher Chl- a content, and moderate disease incidence. However, the white pulp genotype responded more favorably to physical protectants such as T2 and T8, particularly in reducing sunburn and preserving chlorophyll.

### 3.2 Effect of treatments on the stomatal and epicuticular wax structures

**Figure 5:**
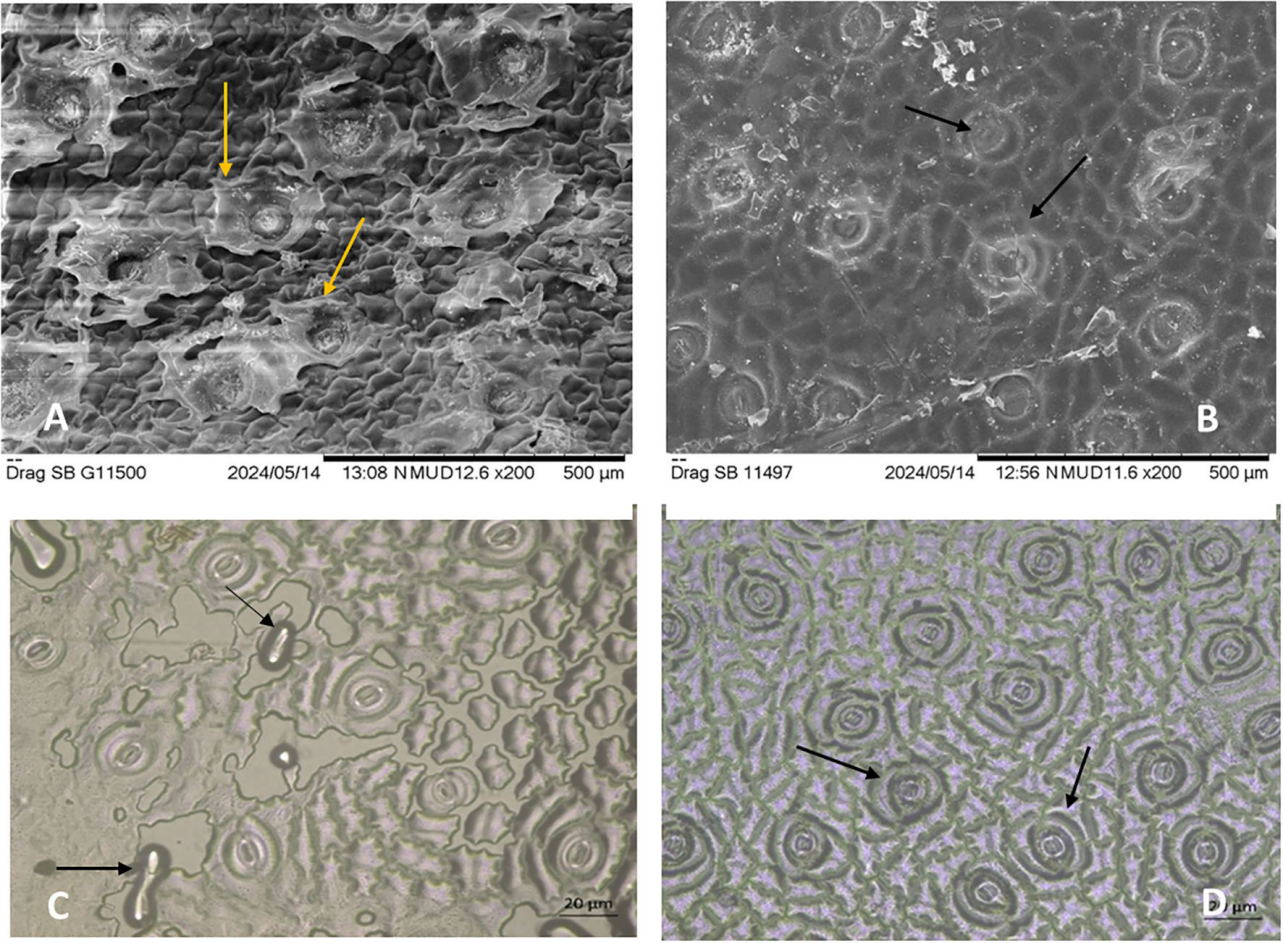
Microscopic analysis of stomatal and epicuticular wax structures in untreated and treated (T8) dragon fruit samples. Untreated samples **(A, C)** show wax disintegration and disrupted stomatal structures. Treated samples **(B, D)** exhibit intact stomatal structures and well-preserved wax layers under SEM indicating the efficacy of the applied treatment in maintaining cuticular integrity. Scale bars: 500 µm **(A, B)**, 20 µm **(C, D)**.

Microscopic analysis of stomatal and epicuticular wax structures in untreated and treated dragon fruit plants (T8) revealed noticeable contrasts in cuticular integrity and stomatal morphology. Scanning electron microscopy (SEM) images demonstrated that untreated samples of both the genotypes (**Figures 5A, C**) exhibited severe wax disintegration, with staggered, flaccid stomata and fractured epicuticular layers, indicative of environmental stress (heat and UV damage). These structural deformities likely compromised the plant’s ability to regulate transpiration and retain moisture, exacerbating sunburn susceptibility. In contrast, treated samples (**Figures 5B, D**) displayed turgid, well-organized stomata and intact epicuticular wax layers, underscoring the protective role of the treatments. The preservation of wax structures is critical for reducing water loss and reflecting excess radiation, suggests that treatments (e.g., kaolin-based neem soap or shade net combinations) mitigated cuticular damage. Notably, the turgidity of stomata in treated plants (**Figure 2D**) implies improved cellular hydration and osmotic regulation, which are vital for stress resilience. These results align with the physiological data, confirming that protective treatments preserve cuticular structures, thereby reducing sunburn incidence and enhancing stress tolerance.

### 3.3 Effect of treatments on phenols, flavonoids and antioxidants

To assess the influence of sunburn mitigating treatments on biochemical profile of red and white pulp genotypes the phenolic content, flavonoid accumulation, and antioxidant capacity was estimated (**Table 2**). These compounds play a crucial role in protecting plants from sunburn and UV damage by acting as UV filters and antioxidants. Notably, total phenol content was generally higher in the white pulp genotype under most treatments, except for T5 (Micronutrients) and T6 (Petroleum Oil), where the red pulp genotype exhibited comparable or slightly elevated levels. The highest phenol concentrations were recorded in the control (T1), with 1.78 mg g^-^¹ in white and 1.72 mg g^-^¹ in red pulp genotype. Treatments typically reduced phenolic accumulation, with the most significant declines observed under T8 (Neem Soap: −34.8% in white, −32.0% in red) and T9 (Neem Soap + Arka Microbial Consortium: −24.7% in white, −21.5% in red). Conversely, T10 (Arka Microbial Consortium) preserved phenol content effectively in the white pulp genotype, while T6 in the red pulp genotype showed slight increase compared to the control.

**Table 2:** Effect of spray treatments on the bioactive compounds of white and red pulp dragon fruit cultivars.

Treatment	Phenols (mg/g FW)		Flavonoids (mg/g FW)		Antioxidants (mg/g FW)	
	White	Red	White	Red	White	Red
T1	1.78a	1.72a	1.11bc	1.25a	2.39bcd	2.61a
T2	1.44de	1.39de	0.83ijk	0.94efgh	2.35bcde	2.12fgh
T3	1.65ab	1.42de	1.06cd	0.98de	2.37bcde	2.48abc
T4	1.52bcd	1.40de	0.94efghi	0.91efghi	2.52ab	2.27defg
T5	1.45de	1.64abc	0.87efghij	1.14abc	2.50ab	2.17efgh
T6	1.47cde	1.73a	0.89efghi	1.88ab	2.47abcd	2.30cdef
T7	1.48bcde	1.35def	0.85hij	0.86ghij	2.45abcd	2.08ghi
T8	1.16g	1.17fg	0.63m	0.68lm	2.34bcde	1.88i
T9	1.34efg	1.35def	0.76jkl	0.70lm	2.41abcd	1.97hi
T10	1.73a	1.34def	0.96defg	0.97def	2.51ab	2.35bcde
T11	1.47cde	1.38de	0.87fghij	0.73klm	2.47abcd	2.07ghi

Values with the same superscript were not significantly different in LSD test *(p< 0.05).*

The red pulp genotype maintained higher flavonoid levels across most treatments. The control (T1) recorded the highest flavonoid content (1.11 mg g^-^¹ in white; 1.25 mg g^-^¹ in red). Among treatments, T6 (Petroleum Oil) increased flavonoids in the red pulp genotype by 50.4%, while T8 caused the largest reductions (−43.2% in white, −45.6% in red). The white pulp genotype exhibited a more gradual and uniform decline in flavonoid content across treatments, whereas the red pulp genotype showed greater variability, reflecting genotype-specific sensitivity.

Antioxidant activity followed trends similar to phenols, with the control (T1) displaying the highest antioxidant content, particularly in the red pulp genotype. The white pulp genotype maintained relatively stable antioxidant levels under different treatments, while the red pulp genotype showed more pronounced fluctuations, with T8 (Kaolin based Neem Soap) causing the steepest decline (−28.0%) aligning with its suppressive effects on phenols and flavonoids. Treatments such as T10 and T6 supported antioxidant retention in both genotypes, while silica (T4) and micronutrient (T5) applications were particularly beneficial in the white pulp genotype. The kaolin-based neem treatments (T8 and T9) significantly reduced all three biochemical traits in both genotypes, whereas microbial and oil-based formulations demonstrated greater potential in preserving or enhancing bioactive metabolite levels.

### 3.4 Effect of treatments on oxidative enzyme activity

The activities of key oxidative stress-related enzymes, including superoxide dismutase (SOD), peroxidase (POD), and catalase (CAT), were significantly influenced by the applied treatments in both white and red pulp genotypes (**Table 3**). SOD activity was highest in the control (T1) and Petroleum Oil treatment (T6), registering 1.63 U mg^-^¹ in the white and 1.61 U mg^-^¹ in the red pulp genotype. This elevated activity in untreated and oil-treated plants suggests higher oxidative stress under these conditions. In contrast, T8 (Kaolin based Neem Soap) significantly reduced SOD activity by 62.6% in white and 31.7% in red, indicating effective stress mitigation. Moderate reductions were observed under T2 and T7, while T5 (Micronutrients) and T3 (Silica) maintained near-control levels in the red pulp genotype.

**Table 3:** Effect of spray treatments on oxidative enzymes and MDA levels in white and red pulp dragon fruit cultivars.

Treatment	SOD (units mg^-^¹ protein)		Peroxidase (units mg^-^¹ protein)		Catalase (units mg^-^¹ protein)		MDA (mg/100g)	
	White	Red	White	Red	White	Red	White	Red
T1	1.63a	1.61a	2.67a	2.52ab	1.30ab	1.42a	5.02a	4.50b
T2	1.13def	1.31bc	2.36bcd	2.28cdef	0.89f	1.18bcd	3.85d	3.80d
T3	1.33b	1.35b	2.30cdef	2.22cdefgh	1.01cdef	1.30ab	4.18c	3.64ef
T4	1.17cdef	1.31bc	2.25cdefg	2.17defgh	0.92ef	1.19abc	3.70e	3.35g
T5	1.54a	1.54a	2.18defgh	2.10fghi	1.16bcd	1.15bcd	3.58f	3.10h
T6	1.62a	1.61a	2.17defgh	2.08ghi	1.20abc	1.17bcd	3.42g	3.04h
T7	1.10ef	1.26bcd	2.14efghi	2.06ghi	0.79fg	1.12bcde	2.81i	2.45k
T8	0.61h	1.10fg	2.01hi	1.93i	0.55h	0.98cdef	2.71j	2.45k
T9	0.95g	1.24bcde	2.53ab	2.43bc	0.66gh	1.20abc	2.52k	2.20l
T10	1.25bcd	1.26bcd	2.23cdefg	2.14efghi	0.96def	1.20abc	2.17lm	2.10m
T11	1.04fg	1.25bcd	2.20defgh	2.33bcde	0.88fg	1.20abc	1.78n	1.43o

Values with the same superscript were not significantly different in LSD test *(p< 0.05).*

POD activity ranged from 2.01 to 2.67 U mg^-^¹ in the white pulp genotype and 1.93 to 2.52 U mg^-^¹ in the red pulp genotype. The control (T1) elicited the highest POD levels in both genotypes (2.67 U mg^-^¹ in white; 2.52 U mg^-^¹ in red), reflecting baseline oxidative stress. Among treatments, T8 showed the lowest activity with reduction of 24.7% and 23.4% in white and red respectively, while T9 (Kaolin+ seaweed+ Arka Neem Soap + Arka Microbial Consortium) partially preserved POD levels, with a minor reduction in the red pulp genotype (−3.6%) compared to the white (−5.2%). Notably, treatments like T2 and T10 exhibited intermediate declines, reflecting partial stress mitigation.

Catalase activity, another critical enzyme in oxidative stress response, followed a similar pattern. Under control, CAT activity was highest in the red pulp genotype (1.42 U mg^-^¹), while the white pulp genotype peaked at 1.30 U mg^-^¹ suggesting genotypic differences. T8 induced the most severe reduction over control 57.7% in white, 31.0% in red, aligning with its suppressive effects on SOD and POD. T5 (Micronutrients) and T3 (Silica) maintained moderate activity in the white pulp genotype, whereas T6 (Petroleum Oil) preserved CAT levels in red (−17.6%).

Among the enzymes, CAT was the most heat-sensitive, showing the largest reductions under T8 compared to the control: a 3.4-fold decrease in the white pulp genotype and a 1.5-fold decrease in the red. SOD followed with 2.7-fold (white) and 1.5-fold (red) declines under T8, while POD was the least affected in both genotypes (≤1.3-fold). Neem Soap (T8) was the most effective treatment for lowering oxidative stress, reducing CAT and SOD activity by 1.5–3.4-fold, whereas T9 (Neem + Microbes) had milder effects (≤1.2-fold). The red pulp genotype exhibited smaller changes (≤1.5-fold vs. white’s ≤3.4-fold) further confirming its greater heat tolerance.

### 3.5 Effect of treatments on membrane system

Malondialdehyde (MDA) levels, reflecting lipid peroxidation, were highest in untreated plants (5.02 mg/100g in white; 4.50 mg/100g in red), confirming severe membrane damage under stress. The most effective treatments were T8 (Neem Soap) and T11 (Brassinosteroids), which reduced MDA by 64.5% (white) and 68.2% (red), demonstrating strong oxidative stress protection. T5 (Micronutrients) and T3 (Silica) also showed efficacy, lowering MDA by 28.7–40.0% in both genotypes. The red pulp genotype consistently exhibited lower MDA than the white across treatments, further supporting its innate stress tolerance. These results align with enzyme data, confirming T8 and T11 as optimal for membrane stability.

### 3.6 Nutrient uptake response to sunburn mitigation treatments

Mineral nutrient analysis was conducted on vegetative parts of red and white dragon fruit genotypes to evaluate the effects of promising sunburn mitigation treatments. Selected treatments viz., shade net (T2), neem soap (T8), and microbial consortium (T9) were compared to the control (T1) to assess their influence on nutrient uptake efficiency. Key findings revealed genotype-specific responses, with significant improvements in macronutrient and micronutrient concentrations under specific treatments.

Nitrogen (N) content was highest in the microbial consortium (T9), with increases of 35% (0.96%) in red and 56% (1.37%) in white pulp genotype compared to the control. Phosphorus (P) uptake was enhanced by 160% under neem soap (T8) in red (0.26%) and 17% under shade net (T2) in white (0.27%). Potassium (K) levels peaked by 129% T9-treated red (2.84%), though a slight reduction (2.31%) was observed in shoot tissues of white pulp genotype. Calcium (Ca) accumulation increased by 21% in T9-treated red (3.34%), while shade net (T2) maintained near-control levels in white pulp genotype. Magnesium (Mg) exhibited contrasting trends, shade net (T2) increased Mg in red by 82% but sharply reduced it in white pulp genotype (90% decrease), suggesting potential genotype-specific antagonism.

Among trace elements, neem soap (T8) significantly boosted manganese (Mn) in red shoots (431.9 ppm) but reduced it in white pulp genotype. Zinc (Zn) levels increased by 151% in T8-treated red pulp genotype, highlighting its role in enzymatic functions while, Boron (B) peaked in shoots of white pulp genotype under shade net (14% increase) supporting structural integrity. Neem soap (T8) caused the largest Na accumulation (143.8%) in shoots of red pulp genotype suggesting possible salt stress. Shade net (T2) increased Na in both genotypes but more prominently in white shoots (51.9%) while the microbial consortium (T9) showed a neutral effect (slight decrease in white shoots).

### 3.7 Correlation, PCA and biplot analysis

**Figure 6:**
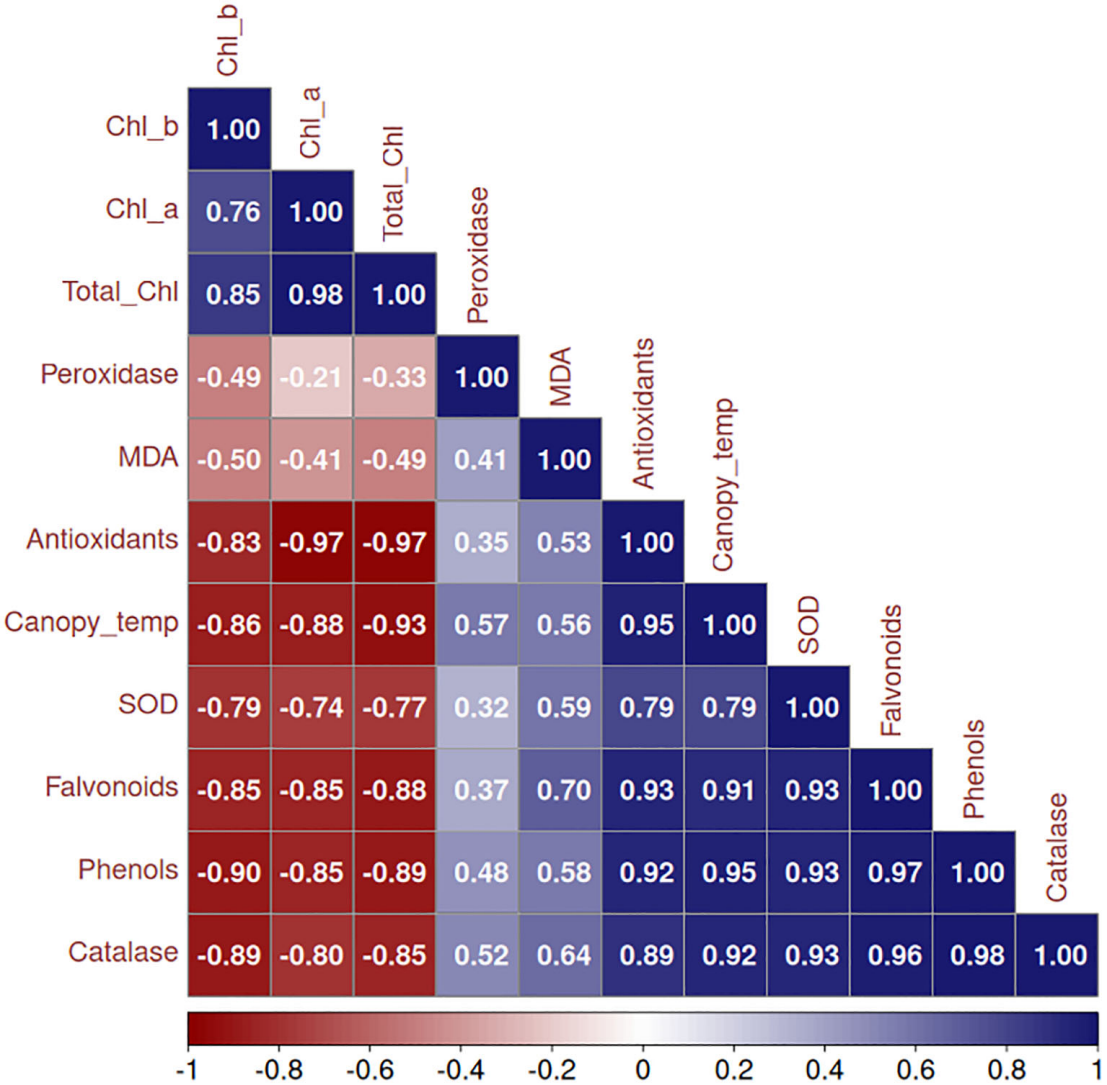
Correlation matrix of physiological and biochemical parameters in dragon fruit under different treatments illustrating the Pearson correlation coefficients, where positive correlations (ranging from 0 to 1) are shown in shades of blue, and negative correlations (ranging from -1 to 0) are shown in shades of red. Darker shades indicate stronger correlations, while lighter shades represent weaker associations.

To elucidate the complex relationships between physiological parameters and treatment effects, we employed three complementary multivariate approaches: Pearson’s correlation analysis to quantify linear associations between variables, Principal Component Analysis (PCA) to reduce dimensionality and identify dominant response patterns, and biplot visualization to examine trait-treatment interactions. These methods collectively revealed the underlying structure of sunburn mitigation mechanisms in dragon fruit. The correlation matrix (**Figure 6**) revealed that chlorophyll components (Chl a, Chl b, and Total Chl) were strongly positively correlated (r=0.8-1.0) while showing negative correlations with canopy temperature (r=-0.9), MDA (r=-0.5), and antioxidants (r=-0.8 to -1.0). MDA exhibited positive correlations with peroxidase activity (r=0.4) and canopy temperature (r=0.6). The antioxidant system components-including SOD, CAT, flavonoids, and phenols-showed strong intercorrelations (r=0.8-0.9), with SOD and CAT being negatively correlated with MDA (r=-0.8 to -0.9). Notably, antioxidants demonstrated the strongest negative correlation with chlorophyll (r=-1.0).

PCA revealed that the first five principal components explained 99.77% of total variation (**Table 1**). PC1 (78.8% variance) was characterized by positive loadings for chlorophyll metrics (Chl a: 0.304; Chl b: 0.308; Total Chl: 0.319) and negative loadings for phenolic compounds (phenols: -0.334; flavonoids: -0.331). PC2 (15.8% variance) was dominated by peroxidase (-0.776) and MDA (-0.397), while PC3 (3.2% variance) highlighted the contrast between MDA (-0.729) and peroxidase (0.467).

**Figure 7:**
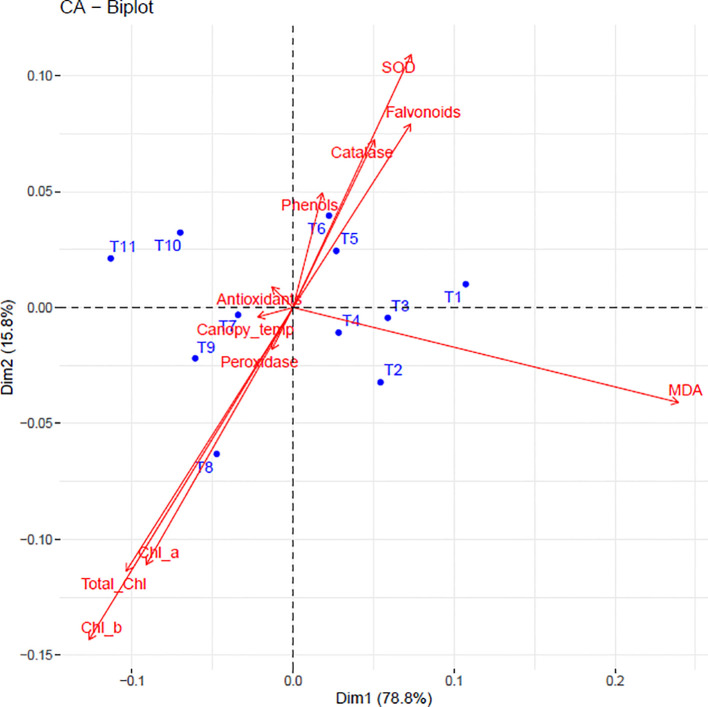
Biplot illustrating the relationships between physiological and biochemical traits and different treatments in dragon fruit. Red vectors represent biochemical and physiological parameters, while blue dots indicate treatments (T1–T11). The direction and length of the arrows indicate the strength and influence of each trait.

The biplot (**Figure 7**) visually confirmed these relationships, with 94.6% variance explained by PC1 and PC2. Treatments clustered according to efficacy: T8 (Kaolin + Seaweed + Neem Soap) and T9 (+ Microbial Consortium) associated with high antioxidant levels and low MDA, while T1 (control) grouped with stress markers. T2 showed strong alignment with chlorophyll preservation, demonstrating treatment-specific response patterns.

### 3.8 Correlogram for the red and white dragon fruit (before vs. after treatment)

**Figure 8:**
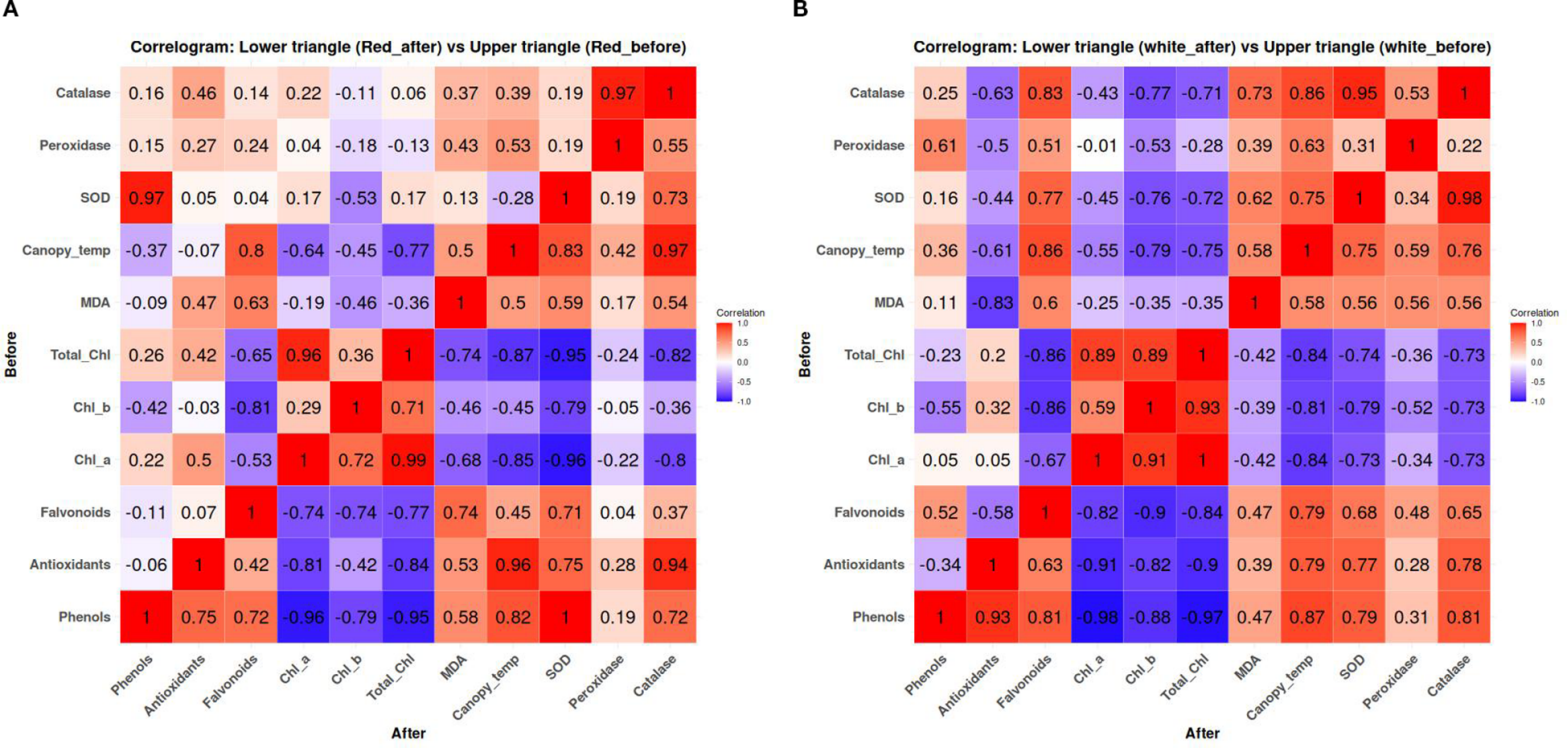
**(a)** Correlogram comparing correlation coefficients of biochemical and physiological traits before (upper triangle) and after (lower triangle) treatment in red pulp dragon fruit. The color gradient represents correlation strength, ranging from blue (negative correlation, -1) to red (positive correlation, +1). **(b)** Correlogram comparing correlation coefficients of biochemical and physiological traits before (upper triangle) and after (lower triangle) treatment in white pulp dragon fruit. The color gradient represents correlation strength, ranging from blue (negative correlation, -1) to red (positive correlation, +1).

Correlogram analysis of red and white dragon fruit genotypes (**Figures 8a, b**) provided critical insights into genotype-dependent treatment efficacy by comparing pre- and post-treatment correlations among physiological traits. This approach revealed fundamental differences in stress adaptation mechanisms between genotypes. In the red pulp genotype, the strong pre-treatment correlation between phenols and antioxidants (r=0.96) decreased post-treatment (r=0.75), concurrent with improved chlorophyll stability as evidenced by the weakened negative correlation with canopy temperature (from r=-0.95 to r=-0.36). Enhanced oxidative stress mitigation was observed through reduced MDA associations with protective compounds and strengthened SOD-catalase coordination (r=0.97 post-treatment). Strong negative correlation (r=-0.77) between canopy temp and catalase implied higher temperature stress decreased antioxidant enzyme activity. This correlation weakened (r=-0.37) after treatment, indicating better enzyme regulation and adaptation to stress. The correlation between SOD and catalase increased after treatment (r=0.97), suggesting enhanced synergy between enzymatic antioxidants in this genotype.

The white pulp genotype exhibited distinct metabolic adjustments, characterized by decreased correlation between antioxidants and flavonoids (from r=0.91 to r=0.63) alongside improved chlorophyll retention (canopy temperature correlation change from r=-0.74 to r=-0.42). Both genotypes demonstrated effective oxidative stress reduction, though through different mechanisms: the red pulp genotype prioritized enzymatic antioxidant synergy, while the white pulp genotype showed greater plasticity in secondary metabolite production. These findings underscore the genotype-specific nature of stress adaptation and highlight the importance of tailored treatment strategies for different dragon fruit genotypes.

## 4 Discussion

Sunburn is a critical challenge in several fruit crops, particularly in regions with hot and arid climates, leading to considerable economic losses and reduced farm productivity (Lal and Sahu, 2017; Fischer et al., 2022). With the depletion of the stratospheric ozone layer and rising global temperatures, UV-B radiation exposure (280–320 nm) is expected to increase, exacerbating sunburn incidence in less tolerant crops like dragon fruit (Munné-Bosch and Vincent, 2019). Reflective particle-based sprays, such as kaolin and neem-based formulations, have emerged as effective strategies to mitigate heat stress by reducing radiation absorption and lowering plant surface temperatures (Reiget al., 2020). Our study evaluated the efficacy of these treatments in red and white pulp dragon fruit genotypes, revealing genotype-specific responses and highlighting the importance of tailored mitigation strategies.

### 4.1 Spray treatments mitigate canopy temperature and sunburn incidence while enhancing chlorophyll content

Our findings demonstrate that the vegetative tissues of dragon fruit are highly sensitive to concurrent exposure to excessive light and high temperatures, resulting in visible sunburn damage (**Figure 2**). This observation is consistent with previous reports on other CAM plants subjected to thermal stress (Raveh et al., 1998; Mizrahi, 2014; Fischer et al., 2022; Doke et al., 2024; Patil et al., 2024). Among the treatments, untreated plants exhibited the highest canopy temperatures (**Table 1**) and the most severe sunburn incidence (78.6–82.1%), highlighting their vulnerability to photothermal stress. In contrast, treatments T2, T8, and T9 significantly reduced sunburn damage, with T2 showing the greatest effectiveness. These results corroborate earlier studies on the use of shade nets in dragon fruit (Patil et al., 2024) and reflective sprays in apple (Faghih et al., 2021; Przybyłko et al., 2025) and grape (Mohamed et al., 2024), which reported similar reductions (10–15%) in sunburn through radiation-reflection mechanisms. Treatments T8 and T9 also showed markedly lower sunburn levels, confirming their strong protective effects. This likely results from the synergistic action of kaolin sprays, neem soap and microbial consortia, which provide a multi-layered defense strategy encompassing physical, biochemical, and hormonal pathways. Kaolin forms a reflective particle film that reduces UV and infrared radiation absorption, thereby lowering tissue temperature and minimizing photo-oxidative stress (del Brio et al., 2024). Neem, when used in combination, further reinforces the cuticle with bioactive compounds such as azadirachtin, which stimulate phenolic biosynthesis and enhance structural integrity against UV-B penetration (El-Beltagi et al., 2024). Additionally, neem provides antifungal protection, reducing the risk of secondary infections (Ali et al., 2025). Since neem was applied in soap form, it likely improved the adhesion of kaolin to the plant surface, enhancing the persistence and effectiveness of the reflective film under field conditions. The microbial consortia comprising of *Pseudomonas fluorescens, Bacillus subtilis, Azospirillum brasilense*, and *Trichoderma harzianum* play a crucial role through phytohormonal modulation (Vishwakarma et al., 2020; Ansabayeva et al., 2025) producing auxins (IAA) to promote tissue repair, abscisic acid (ABA) to regulate stomatal conductance and improve water retention (especially vital for CAM plants like dragon fruit), and ACC deaminase to suppress stress-induced ethylene synthesis that accelerates senescence (Cheynier et al., 2013; Maheshwari et al., 2015). Beneficial microbes also enhance the activity of antioxidant enzymes such as superoxide dismutase (SOD) and catalase (CAT), which help scavenge reactive oxygen species (ROS) generated under heat stress (Ahemad and Kibret, 2014). Together, these components work synergistically- kaolin mitigates the initial radiation load, neem enhances structural defenses, and PGPRs (plant growth-promoting rhizobacteria) prime the plant’s systemic resilience via hormonal regulation and redox balance. This integrated, tripartite strategy is particularly effective for sunburn-sensitive white-fleshed dragon fruit cultivars, whose genetic susceptibility is counterbalanced by the combined physical barrier (kaolin), biochemical reinforcement (neem), and physiological adaptation (PGPR-mediated stress priming). The inclusion of stress-adapted PGPR strains selected for their capacity to modulate ABA/GA ratios and induce heat-shock proteins further strengthens field performance, making this integrated approach superior to single-component interventions for maintaining productivity under intensifying climatic stressors. Epicuticular wax integrity, critical for UV and PAR scattering, was preserved in treated plants, as evidenced by SEM imaging (**Figure 5**). The protective role of epicuticular wax against photothermal stress has been extensively documented (Domanda et al., 2024). Untreated samples showed fractured wax layers and flaccid stomata, while treated plants maintained turgid stomata and intact cuticles, enhancing water retention and stress resilience. Similar structural preservation has been reported in kaolin-treated olive leaves (Rotondi et al., 2025) and neem-protected mango fruits (Sarmin et al., 2018). The red pulp genotype, with its thicker wax coatings and sunken stomata, exhibited superior thermal tolerance, consistent with findings by Raveh et al. (1998) and subsequent studies on cactus morphology (Nobel, 2003). Chlorophyll degradation (yellowing), a marker of sunburn damage, was markedly reduced in treated plants. This protection against chlorophyll loss has been similarly observed in kaolin-treated grapevines (Dinis et al., 2016) and shade net covered apple trees (Iglesias and Alegre, 2006). T8 (neem soap) increased Chl-a by 71.4% in the white pulp genotype, while the red pulp genotype maintained higher baseline Chl-a levels across treatments. This differential response corroborates findings in other pigmented versus non-pigmented fruit varieties (Gould et al., 2010). Pigmented genotypes often exhibit enhanced stress tolerance due to antioxidant pigments such as betacyanins. A study in *Amaranthus tricolor* L. demonstrated that the chromatic cultivar, with higher betacyanin content, retained more chlorophyll, accumulated less H_2_O_2_, and exhibited greater PSII stability under high-temperature stress compared to the green cultivar (Shu et al., 2009). Similarly, the red pulp genotype in our study likely benefits from pigment-mediated photoprotection, while the white pulp genotype, lacking such pigments, showed greater responsiveness to external protection via neem soap. Our findings align with reports that kaolin and neem extracts protect chloroplast integrity by mitigating ROS-induced thylakoid damage (Zahedi et al., 2020; Naz et al., 2022; Hameed et al., 2023). Shade net protection (T2) further reduced chlorophyll loss by moderating light intensity, as observed in studies by Chang et al. (2016) and Patil et al., 2024 in dragon fruit and other fruit crops (Díaz-Pérez, 2014; Bastías et al., 2012). Microclimate modification under shade nets has been shown to reduce direct light exposure by 30–50% while increasing relative humidity (Mahmood et al., 2018), thereby creating more favorable conditions for photosynthesis and reducing photoinhibition (Sofo et al., 2009).

### 4.2 Biochemical and enzymatic defense mechanisms

Secondary metabolites such as phenols and flavonoids play a crucial role in UV screening and ROS scavenging (Agati et al., 2012). Our findings are consistent with established reports showing that stress conditions stimulate phenolic biosynthesis (Kumar et al., 2023), as evidenced by elevated levels in untreated controls. Treatment-induced reductions in these compounds suggest effective stress mitigation. Notably, the red pulp genotype maintained higher baseline levels of phenols, flavonoids, and antioxidant activity, reflecting inherent thermal tolerance—consistent with cactus stress physiology (Raveh et al., 1998) and anthocyanin-mediated photoprotection in pigmented fruits (Mishra et al., 2025). Betacyanins, while primarily localized in fruit peel and pulp, can also be synthesized in vegetative tissues under stress and may contribute to enhanced protection in red pulp genotypes. This putative role, potentially governed by genotype-specific regulation, warrants further investigation through histochemical and transcriptomic approaches. In contrast, the white pulp genotype exhibited more dynamic shifts in phenolic metabolites and antioxidant enzymes, suggesting greater reliance on inducible defense systems. This response implicates stress-responsive hormonal pathways, particularly those involving ABA, JA, and SA, which regulate phenylpropanoid metabolism (Verma et al., 2016). The sharp decline in SOD (62.6%) and CAT (57.7%) activities under T8 treatment in white pulp plants further highlights that less reactive oxygen species (ROS) is produced, which reduces their dependence on enzymatic antioxidant systems, consistent with earlier reports (Foyer and Noctor, 2005; Hamdy et al., 2022). The lipid peroxidation data, with MDA reductions up to 68.2% under optimal treatments, provide strong evidence for membrane stabilization effects. These results not only confirm neem’s protective role (Naz et al., 2022; Hameed et al., 2023) but also demonstrate its effectiveness in perennial fruit crops under field conditions. The correlation between MDA reduction and improved chlorophyll retention (r=-0.5) supports the growing recognition of membrane integrity as a key determinant of photosynthetic efficiency under stress (Shakya, 2023). Several mechanisms may explain the superior performance of neem-based treatments such as physical protection from UV radiation through particle film formation, direct antioxidant activity (Brahmachari, 2004), antimicrobial effects reducing secondary infections (Alzohairy, 2016) and hormonal modulation of stress responses (Subapriya and Nagini, 2005). The genotype-specific responses have important implications for both breeding and cultivation. The red genotype’s betalain-mediated constitutive defense (Shu et al., 2009) suggests potential for selecting pigment-rich cultivars in stress-prone environments. Conversely, the white genotype’s responsiveness to particle film treatments (Naz et al., 2022; Hameed et al., 2023) supports targeted agronomic interventions. These findings collectively expand our understanding of secondary metabolite regulation in perennial fruit crops under abiotic stress (Csepregi and Hideg, 2018), while providing practical solutions for crop management.

### 4.3 Nutrient uptake and physiological response to sunburn mitigation treatments

The comprehensive analysis of nutrient dynamics in this study reveals fundamental differences in how the red and white pulp dragon fruit genotypes respond to various sunburn mitigation treatments, providing important insights for stress management in perennial fruit crops (**Table 4**). The observed nutrient uptake patterns demonstrate that while untreated control plants (T1) maintained basic nutritional sufficiency, their significantly lower K levels compared to plants receiving the T9 treatment (Kaolin + Seaweed + Neem Soap + Arka Microbial Consortium) clearly indicate that natural soil nutrient availability becomes inadequate under stress conditions (Sardans and Peñuelas, 2021). This finding has critical implications for orchard management, particularly in climate-stressed environments where plants face combined abiotic pressures. The red pulp genotype exhibited particularly strong enhancement of potassium uptake (129% increase under T9), which likely contributes significantly to its observed stress tolerance. Potassium plays multiple well-documented physiological roles that could explain this genotype’s resilience, including its function in stomatal regulation (Wang et al., 2014), activation of crucial enzymes, and mitigation of oxidative stress (Hasanuzzaman et al., 2018). The mechanisms behind this improved K acquisition appear multifaceted, involving both direct and indirect pathways. Microbial-mediated nutrient mobilization through the Arka Microbial Consortium probably enhanced rhizosphere K availability through organic acid secretion and soil structure modification (Selvakumar et al., 2012), effects that have been similarly documented in other crop systems (Rouphael and Colla, 2018). Simultaneously, the treatment’s seaweed components likely stimulated root growth through their cytokinin and auxin-like compounds (Khan et al., 2009), while neem constituents improved root efficiency (Brahmachari, 2004; Lokanadhan et al., 2012). The kaolin component contributed by creating a more favorable root zone environment through improved water use efficiency and temperature moderation (Glenn et al., 2010). In striking contrast, the white pulp genotype showed a distinctly different response pattern, with particular sensitivity to phosphorus (P) availability. This genotype displayed a 17% increase in P uptake under T2 treatment (Kaolin + Green Shade Net), likely resulting from shade-induced modifications to soil temperature regimes that promoted microbial P mineralization (Mooshammer et al., 2017). However, this P responsiveness came at a substantial cost, with a dramatic 90% reduction in K levels. Several interacting factors probably contribute to this trade-off: reduced transpiration under shaded conditions may have decreased mass flow-driven K transport to roots (Gajdanowicz et al., 2011), while light quality changes could have affected expression of potassium transporters (Ahammed et al., 2022). Additionally, the shade environment likely triggered changes in root architecture and allocation patterns (Poorter et al., 2012), potentially favoring P-acquiring structures over those optimized for K uptake. These genotype-specific responses highlight fundamental differences in nutrient use efficiency (White et al., 2013) and stress adaptation strategies. The red genotype’s ability to maintain superior K homeostasis across treatments suggests a more robust ion regulation system, while the white genotype’s greater plasticity in P acquisition indicates a different evolutionary strategy for nutrient management. The superior performance of the integrated T9 treatment across all measured nutrients (35-56% increase in N, up to 160% in P, and 129% in K) demonstrates that combined approaches addressing multiple stress factors simultaneously-physical protection, biostimulation, and microbial enhancement-offer the most effective solution for stress mitigation in dragon fruit cultivation. These findings have immediate practical applications for precision orchard management, particularly in matching treatment formulations to genotype-specific nutritional requirements and recognizing the critical importance of potassium nutrition in shaded growing systems. The results advance our understanding of nutrient-mediated stress responses while providing evidence-based strategies for improving crop resilience in changing climates.

**Table 4:** Effect of promising spray treatments on mineral uptake in white and red pulp dragon fruit cultivars.

Treatment	N (%)		P (%)		K (%)		Ca (%)		Mg (%)	
	White	Red	White	Red	White	Red	White	Red	White	Red
**T1**	0.88d	0.71ef	0.23ab	0.1d	2.57ab	1.24c	3.34a	2.75bc	1.02ab	0.44c
**T2**	1.09b	0.76e	0.27a	0.23ab	1.33c	1.33c	3.26a	2.77bc	0.10ab	0.80b
**T8**	1.31a	0.68f	0.18bc	0.26a	1.57c	2.66ab	2.63c	3.15a	0.11a	0.12a
**T9**	1.37a	0.96c	0.17c	0.15c	2.31b	2.84a	3.06ab	3.34a	0.06b	0.10ab

Values with the same superscript were not significantly different in LSD test *(p >< 0.05)*

**Table:** 

Treatment	Mn (ppm)		Zn (ppm)		B (ppm)		Na (%)		S (%)		Fe (ppm)	
	White	Red	White	Red	White	Red	White	Red	White	Red	White	Red
**T1**	248.20c	224.80d	18.70c	13.00d	118.50b	99.60c	0.27b	0.16d	0.12a	0.06b	93.80abc	79.40c
**T2**	240.50c	248.10c	26.00b	17.30cd	134.60a	112.40b	0.41a	0.18cd	0.10ab	0.10ab	97.30a	92.40abc
**T8**	139.80f	431.90a	19.00c	32.60a	95.40c	116.50b	0.35a	0.39a	0.11a	0.12a	81.70bc	95.50ab
**T9**	166.30e	391.70b	16.80cd	27.40b	99.60c	114.50b	0.26b	0.23bc	0.06b	0.10ab	94.40ab	94.00abc

Values with the same superscript were not significantly different in LSD test *(p< 0.05).*

### 4.4 Multivariate analysis of biochemical and physiological traits under sunburn mitigation treatments

The multivariate analysis provided a comprehensive understanding of the complex interactions between physiological and biochemical traits in dragon fruit under sunburn stress and mitigation treatments. Our results demonstrate that chlorophyll content exhibited strong negative correlations with canopy temperature (r=-0.9) and MDA (r=-0.5), reinforcing its role as a key indicator of oxidative stress mitigation. This aligns with previous studies in heat-stressed crops where chlorophyll preservation was linked to reduced membrane damage (Zahra et al., 2023). The positive correlation between MDA and peroxidase activity (r=0.4) suggests that lipid peroxidation triggers peroxidase-mediated defense mechanisms, consistent with findings in drought-stressed plants (Gill and Tuteja, 2010). Additionally, the strong intercorrelation among antioxidant components (SOD, CAT, phenols, flavonoids; r=0.8–0.9) indicates a tightly regulated, synergistic defense network, as reported in other ROS-scavenging systems (Hasanuzzaman et al., 2018). PCA revealed that PC1 (78.8% variance) was dominated by chlorophyll metrics and phenolic compounds, highlighting their opposing roles in stress response, chlorophyll as a stability marker and phenols as stress-induced antioxidants. Under stress conditions, chlorophyll degradation occurs due to reactive oxygen species (ROS) accumulation, while phenolic compounds are synthesized as part of the antioxidant defense system (Agati et al., 2012). This inverse relationship was clearly captured by the first principal component in our correspondence analysis, consistent with observations in other perennial crops where kaolin applications similarly decoupled photosynthetic traits from stress markers (Dinis et al., 2016; Glenn et al., 2010). Treatment-specific responses further validated this relationship. The integrated T9 treatment (Kaolin 5% + Seaweed 0.5% + Arka Neem soap 0.5% + Arka Microbial Consortium 0.5%) effectively reduced oxidative stress, as evidenced by its association with both chlorophyll preservation and moderate phenolic levels in the biplot. This balanced response mirrors findings from grapevine studies where combined physical and biochemical treatments optimized stress adaptation (Dinis et al., 2016). In contrast, T2 (Kaolin + Shade net) showed the strongest association with chlorophyll retention, supporting its role in light stress mitigation (Chang et al., 2016; Patil et al., 2024), while T8 and T9 clustered with enhanced antioxidant capacity and reduced lipid peroxidation (MDA). Control (T1) and petroleum oil (T6) treatments aligned with elevated oxidative damage markers, consistent with reports of untreated crops under heat stress (Racsko and Schrader, 2012). These results demonstrate that the chlorophyll-phenolic opposition represents a core physiological trade-off in stress responses, and that targeted treatments can modulate this relationship to improve plant resilience. The ability of certain treatments (particularly T9) to simultaneously maintain photosynthetic function while managing oxidative stress highlights the potential of integrated approaches for sustainable sunburn mitigation in dragon fruit cultivation. Correlogram analysis further elucidated genotype-specific adaptations. In the red pulp genotype, the weakened post-treatment correlation between phenols and antioxidants (r=0.96 reduced to 0.75) suggests a shift from chemical to enzymatic defenses (SOD-CAT synergy: r=0.97). This aligns with studies on pigmented fruits, where anthocyanins supplement antioxidant capacity under stress (Gould et al., 2002). Conversely, the white pulp genotype showed greater reliance on phenolic plasticity, as evidenced by retained flavonoid-antioxidant correlations (r=0.63 post-treatment). Such divergence underscores the need for genotype-specific mitigation strategies, as proposed for other crops with varietal stress tolerance differences (Agati et al., 2012).

## 5 Conclusion

This study demonstrates that kaolin-based protective sprays, particularly when combined with neem soap and microbial consortia, effectively mitigate sunburn stress in dragon fruit by reducing canopy temperature, preserving chlorophyll content, and enhancing antioxidant defenses. The red-fleshed cultivar exhibited greater inherent tolerance to sunburn compared to the white-fleshed cultivar, which responded more favorably to physical protection methods. These treatments work synergistically to improve plant resilience through multiple mechanisms, including radiation reflection, oxidative stress reduction, and enhanced nutrient uptake. The findings provide practical, scalable solutions for sunburn management in dragon fruit cultivation, particularly in regions facing increasing solar radiation and temperature extremes. Future studies should explore the long-term effects of these treatments under field conditions, investigate their impact on fruit quality and yield parameters, and examine potential synergistic effects with other stress-mitigation strategies. Additionally, molecular approaches could elucidate the genetic basis of differential sunburn tolerance between cultivars, informing breeding programs for more resilient varieties.

## Data Availability

The original contributions presented in the study are included in the article/**Supplementary Material**. Further inquiries can be directed to the corresponding authors.

## References

[B1] Afonso S. Gonçalves M. Rodrigues M. Martinho F. Amado V. Rodrigues S.. (2025). Conventional vs. photoselective nets: Impacts on tree physiology, yield, fruit quality and sunburn in “Gala” apples grown in Mediterranean climate. Agronomy 15, 1812. doi: 10.3390/agronomy15081812

[B2] Agati G. Azzarello E. Pollastri S. Tattini M. (2012). Flavonoids as antioxidants in plants: Location and functional significance. Plant Sci. 196, 67–76. doi: 10.1016/j.plantsci.2012.07.014, PMID: 23017900

[B3] Ahammed G. J. Chen Y. Liu C. Yang Y. (2022). Light regulation of potassium in plants. Plant Physiol. Biochem. 170, 316–324. doi: 10.1016/j.plaphy.2021.12.012, PMID: 34954566

[B4] Ahemad M. Kibret M. (2014). Mechanisms and applications of plant growth promoting rhizobacteria: Current perspective. J. King Saud University-Science 26, 1–20. doi: 10.1016/j.jksus.2013.05.001

[B5] Ali J. Hussain A. Abbas J. (2025). *In vitro* control of post-harvest fruits rot pathogenic fungi using *Azadirachta indica* (Neem) seeds and leaves extracts. Bull. Biol. Allied Sci. Res. 1, 102. doi: 10.54112/bbasr.v2025i1.102

[B6] Ali O. Ramsubhag A. Jayaraman J. (2021). Biostimulant properties of seaweed extracts in plants: Implications towards sustainable crop production. Plants 10, 531. doi: 10.3390/plants10030531, PMID: 33808954 PMC8000310

[B7] Al-Saif A. M. Mosa W. F. Saleh A. A. Ali M. M. Sas-Paszt L. Abada H. S.. (2022). Yield and fruit quality response of pomegranate (*Punica granatum*) to foliar spray of potassium, calcium and kaolin. Horticulturae 8, 946. doi: 10.3390/horticulturae8100946

[B8] Alzohairy M. A. (2016). Therapeutics role of *Azadirachta indica* (Neem) and their active constituents in diseases prevention and treatment. Evidence-Based Complementary Altern. Med., 7382506. doi: 10.1155/2016/7382506, PMID: 27034694 PMC4791507

[B9] Amogi B. R. Ranjan R. Pukrongta N. Khot L. R. Sallato B. V. Mogollón M. R.. (2025). Localized sensing data-driven efficacy evaluation of heat stress mitigation techniques in ‘Honeycrisp’ apple cultivar. Scientia Hortic. 341, 113992. doi: 10.1016/j.scienta.2025.113992

[B10] Ansabayeva A. Makhambetov M. Rebouh N. Y. Abdelkader M. Saudy H. S. Hassan K. M.. (2025). Plant growth-promoting microbes for resilient farming systems: Mitigating environmental stressors and boosting crops productivity—A review. Horticulturae 11, 260. doi: 10.3390/horticulturae11030260

[B11] Apak R. Güçlü K. Özyürek M. Karademir S. E. (2004). Novel total antioxidant capacity index for dietary polyphenols and vitamins C and E, using their cupric ion reducing capability in the presence of neocuproine: CUPRAC method. J. Agric. Food Chem. 52, 7970–7981. doi: 10.1021/jf048741x, PMID: 15612784

[B12] Arivalagan M. Karunakaran G. Roy T. K. Dinsha M. Sindhu B. C. Shilpashree V. M. (2021). Biochemical and nutritional characterization of dragon fruit (*Hylocereus* species). Food Chemistry 353, 129426. doi: 10.1016/j.foodchem.2021.129426, PMID: 33774520

[B13] Ayangbenro A. S. Babalola O. O. (2021). Reclamation of arid and semi-arid soils: The role of plant growth-promoting archaea and bacteria. Curr. Plant Biol. 25, 100173. doi: 10.1016/j.cpb.2021.100173

[B14] Bastías R. M. Manfrini L. Grappadelli L. C. (2012). Exploring the potential use of photo-selective nets for fruit growth regulation in apple. Scientia Hortic. 143, 101–108. doi: 10.1016/j.scienta.2012.06.011

[B15] Benzie I. F. Strain J. J. (1996). The ferric reducing ability of plasma (FRAP) as a measure of “antioxidant power”: The FRAP assay. Analytical Biochem. 239, 70–76. doi: 10.1006/abio.1996.0292, PMID: 8660627

[B16] Bita C. E. Gerats T. (2013). Plant tolerance to high temperature in a changing environment: Scientific fundamentals and production of heat stress-tolerant crops. Front. Plant Sci. 4. doi: 10.3389/fpls.2013.00273, PMID: 23914193 PMC3728475

[B17] Boini A. Bortolotti G. Morandi B. (2024). “Orchard management changes solar radiation profiles, influencing apple sunburn,” in EHC2024: International Symposium on Robotics, Mechanization and Smart Horticulture Acta Hortic., Vol. 1433. 155–160. doi: 10.17660/ActaHortic.2025.1433.19

[B18] Brahmachari G. (2004). Neem—An omnipotent plant: A retrospection. ChemBioChem 5, 408–421. doi: 10.1002/cbic.200300749, PMID: 15185362

[B19] Brand-Williams W. Cuvelier M. E. Berset C. (1995). Use of a free radical method to evaluate antioxidant activity. LWT-Food Sci. Technol. 28, 25–30. doi: 10.1016/S0023-6438(95)80008-5

[B20] Brito C. Dinis L. T. Moutinho-Pereira J. Correia C. (2019). Kaolin, an emerging tool to alleviate the effects of abiotic stresses on crop performance. Scientia Hortic. 250, 310–316. doi: 10.1016/j.scienta.2019.02.070

[B21] Chang P. T. Hsieh C. C. Jiang Y. L. (2016). Responses of ‘Shih Huo Chuan’ pitaya (*Hylocereus polyrhizus* (Weber) Britt. & Rose) to different degrees of shading nets. Scientia Hortic. 198, 154–162. doi: 10.1016/j.scienta.2015.11.024

[B22] Cheynier V. Comte G. Davies K. M. Lattanzio V. Martens S. (2013). Plant phenolics: Recent advances on their biosynthesis, genetics, and ecophysiology. Plant Physiol. Biochem. 72, 1–20. doi: 10.1016/j.plaphy.2013.05.009, PMID: 23774057

[B23] Chien Y. Chang J. (2019). Net houses effects on microclimate, production, and plant protection of white-fleshed pitaya. HortScience 54, 692–700. doi: 10.21273/HORTSCI13850-18

[B24] Chu Y. C. Chang J. C. (2020). High temperature suppresses fruit/seed set and weight, and cladode regreening in red-fleshed ‘Da Hong’ pitaya (*Hylocereus polyrhizus*) under controlled conditions. HortScience 55, 1259–1264. doi: 10.21273/HORTSCI15022-20

[B25] Contreras C. Zoffoli J. Alcalde J. Ayala M. (2008). Evolution of sunburn damage on ‘Granny Smith’ apples during storage. Ciencia e Investigación Agraria 35, 147–157. doi: 10.4067/S0718-16202008000200003

[B26] Cruz A. L. A. Montalvo I. A. G. Pérez D. M. Santiago A. D. P. Medina M. A. S. (2025). Use of microbial consortia in agriculture as an alternative for achieving sustainable agriculture. Ciencia Latina Rev. Científica Multidisciplinar 9, 872–887. doi: 10.37811/cl_rcm.v9i2.16893

[B27] Csepregi K. Hideg É. (2018). Phenolic compound diversity explored in the context of photo-oxidative stress protection. Phytochemical Anal. 29, 129–136. doi: 10.1002/pca.2734, PMID: 28895264

[B28] Danko R. Pavloušek P. Kapłan M. Klimek K. E. (2024). Conception, consequences and design of cool climate viticulture training systems. Agriculture 14, 1966. doi: 10.3390/agriculture14111966

[B29] Dawood M. F. Latef A. A. H. A. (2023). “ *Allium cepa* under stressful conditions,” in Medicinal Plant Responses to Stressful Conditions. (CRC Press: Boca Raton, FL: Abingdon, Oxon), 1–20.

[B30] del Brio J. Castro A. Curetti M. Venturino A. Raffo M. D. (2024). Lyphophilic coat sprays reduce sun damage and improve fruit quality in ‘Beurré D’Anjou’ pears. Scientia Hortic. 336, 113363. doi: 10.1016/j.scienta.2024.113363

[B31] Díaz-Pérez J. C. (2014). Bell pepper (*Capsicum annum* L.) crop as affected by shade level: Fruit yield, quality, and postharvest attributes, and incidence of phytophthora blight (caused by *Phytophthora capsici* Leon.). HortScience 49, 891–900. doi: 10.21273/HORTSCI.49.7.891

[B32] Dinis L. T. Bernardo S. Conde A. Pimentel D. Ferreira H. Félix L.. (2016). Kaolin exogenous application boosts antioxidant capacity and phenolic content in berries and leaves of grapevine under summer stress. J. Plant Physiol. 191, 45–53. doi: 10.1016/j.jplph.2015.12.005, PMID: 26717011

[B33] Do V. G. Lee Y. Park J. Win N. M. Kwon S. I. Yang S.. (2024). Heat stress and water irrigation management effects on the fruit color and quality of ‘Hongro’ apples. Agriculture 14, 761. doi: 10.3390/agriculture14050761

[B34] Doke A. Kakade V. D. Patil R. A. Morade A. S. Chavan S. B. Salunkhe V. N.. (2024). Enhancing plant growth and yield in dragon fruit (*Hylocereus undatus*) through strategic pruning: A comprehensive approach for sunburn and disease management. Scientia Hortic. 337, 113562. doi: 10.1016/j.scienta.2024.113562

[B35] Domanda C. Paradiso V. M. Migliaro D. Pappaccogli G. Failla O. Rustioni L. (2024). Epicuticular waxes: A natural packaging to deal with sunburn browning in white grapes. Scientia Hortic. 328, 112856. doi: 10.1016/j.scienta.2024.112856

[B36] El-Beltagi H. S. Ragab M. Osman A. El-Masry R. A. Alwutayd K. M. Althagafi H.. (2024). Biosynthesis of zinc oxide nanoparticles via neem extract and their anticancer and antibacterial activities. PeerJ 12, e17588. doi: 10.7717/peerj.17588, PMID: 38948224 PMC11212640

[B37] Faghih S. Zamani Z. Fatahi R. Omidi M. (2021). Influence of kaolin application on most important fruit and leaf characteristics of two apple cultivars under sustained deficit irrigation. Biol. Res. 54. doi: 10.1186/s40659-021-00328-4, PMID: 33407933 PMC7789529

[B38] Fischer G. Orduz-Rodríguez J. O. Amarante C.V.T.do. (2022). Sunburn disorder in tropical and subtropical fruits. A review. Rev. Colombiana Cienc. Hortícolas 16, e15703. doi: 10.17584/rcch.2022v16i3.15703

[B39] Flénet F. Kiniry J. R. Board J. E. Westgate M. E. Reicosky D. C. (1996). Row spacing effects on light extinction coefficients of corn, sorghum, soybean, and sunflower. Agron. J. 88, 185–190. doi: 10.2134/agronj1996.00021962008800020014x

[B40] Foyer C. H. Noctor G. (2005). Redox homeostasis and antioxidant signaling: A metabolic interface between stress perception and physiological responses. Plant Cell 17, 1866–1875. doi: 10.1105/tpc.105.033589, PMID: 15987996 PMC1167537

[B41] Gajdanowicz P. Michard E. Sandmann M. Rocha M. Corrêa L. G. G. Ramírez-Aguilar S. J.. (2011). Potassium (K+) gradients serve as a mobile energy source in plant vascular tissues. Proc. Natl. Acad. Sci. 108, 864–869. doi: 10.1073/pnas.1009777108, PMID: 21187374 PMC3021027

[B42] Gambetta J. M. Holzapfel B. P. Stoll M. Friedel M. (2021). Sunburn in grapes: A review. Front. Plant Sci. 11. doi: 10.3389/fpls.2020.604691, PMID: 33488654 PMC7819898

[B43] Gill S. S. Tuteja N. (2010). Reactive oxygen species and antioxidant machinery in abiotic stress tolerance in crop plants. Plant Physiol. Biochem. 48, 909–930. doi: 10.1016/j.plaphy.2010.08.016, PMID: 20870416

[B44] Glenn D. M. Cooley N. Walker R. Clingeleffer P. Shellie K. (2010). Impact of kaolin particle film and water deficit on wine grape water use efficiency and plant water relations. HortScience 45, 1178–1187. doi: 10.21273/HORTSCI.45.8.1178

[B45] Gould K. S. Dudle D. A. Neufeld H. S. (2010). Why some stems are red: Cauline anthocyanins shield photosystem II against high light stress. J. Exp. Bot. 61, 2707–2717. doi: 10.1093/jxb/erq106, PMID: 20400528 PMC2882266

[B46] Gould K. S. McKelvie J. Markham K. R. (2002). Do anthocyanins function as antioxidants in leaves? Imaging of H2O2 in red and green leaves after mechanical injury. Plant Cell Environ. 25, 1261–1269. doi: 10.1046/j.1365-3040.2002.00905.x

[B47] Hamdy A. E. Abdel-Aziz H. F. El-Khamissi H. AlJwaizea N. I. El-Yazied A. A. Selim S.. (2022). Kaolin improves photosynthetic pigments, and antioxidant content, and decreases sunburn of mangoes: Field study. Agronomy 12, 1535. doi: 10.3390/agronomy12071535

[B48] Hameed S. Atif M. Perveen S. (2023). Role of gibberellins, neem leaf extract, and serine in improving wheat growth and grain yield under drought-triggered oxidative stress. Physiol. Mol. Biol. Plants 29, 1675–1691. doi: 10.1007/s12298-023-01340-6, PMID: 38162918 PMC10754809

[B49] Hasanuzzaman M. Bhuyan M. B. Nahar K. Hossain M. S. Mahmud J. A. Hossen M. S.. (2018). Potassium: A vital regulator of plant responses and tolerance to abiotic stresses. Agronomy 8, 31. doi: 10.3390/agronomy8030031

[B50] Heath R. L. Packer L. (2022). Reprint of: Photoperoxidation in isolated chloroplasts I. Kinetics and stoichiometry of fatty acid peroxidation. Arch. Biochem. Biophysics 726, 109248. doi: 10.1016/j.abb.2022.109248, PMID: 35667910

[B51] Iglesias I. Alegre S. (2006). The effect of anti-hail nets on fruit protection, radiation, temperature, quality and profitability of ‘Mondial Gala’ apples. J. Appl. Horticulture 8, 91–100. doi: 10.37855/jah.2006.v08i02.22

[B52] Inskeep W. P. Bloom P. R. (1985). Extinction coefficients of chlorophyll a and b in N, N-dimethylformamide and 80% acetone. Plant Physiology 77, 483–485. doi: 10.1104/pp.77.2.483, PMID: 16664080 PMC1064541

[B53] Jbir-Koubaa R. Charfeddine S. Ellouz W. Saidi M. N. Drira N. Gargouri-Bouzid R.. (2015). Investigation of the response to salinity and to oxidative stress of interspecific potato somatic hybrids grown in a greenhouse. Plant Cell Tissue Organ Culture 120, 933–947. doi: 10.1007/s11240-014-0652-8

[B54] Kakade V. D. Boraiah K. M. Salunkhe V. S. Nangare D. D. Chavan S. B. Wakchaure G. C.. (2023). Emerging technologies for enhancing productivity and quality of dragon fruit in water scarce and degraded areas. *Technical Folder no. 2023/56* . (Baramati: ICAR-NIASM).

[B55] Karunakaran G. Arivalagan M. Sriram S. (2019). “Dragon fruit country report from India,” in Workshop “Dragon fruit network: Marketing and the whole value chain. (Vietnam: FFTC and VAAS-SOFRI), 105–112.

[B56] Karunakaran G. Sakthivel T. Arivalagan M. Reddy D. L. Tripathi P. C. Kalaivanan D. (2024). Investigation on promising progenies of Dragon fruit (*Hylocereus* spp.). J. Hortic. Sci. 19, 2633. doi: 10.24154/jhs.v19i1.2633

[B57] Khan W. Rayirath U. P. Subramanian S. Jithesh M. N. Rayorath P. Hodges D. M.. (2009). Seaweed extracts as biostimulants of plant growth and development. J. Plant Growth Regul. 28, 386–399. doi: 10.1007/s00344-009-9103-x

[B58] Kishore K. Sangeetha G. Rupa T. R. Ganeshmurthy A. N. Samant D. Acharya G. C.. (2025). Assessment of mango-based intercropping systems for productivity, resource use efficiency and environmental sustainability in tropical region of India. Next Sustainability 5, 100121. doi: 10.1016/j.nxsust.2025.100121

[B59] Krasnow M. N. Matthews M. A. Smith R. J. Benz J. Weber E. Shackel K. A. (2010). Distinctive symptoms differentiate four common types of berry shrivel disorder in grape. California Agric. 64, 155–158. doi: 10.3733/ca.v064n03p155

[B60] Kumar G. Nanda S. Singh S. K. Kumar S. Singh D. Singh B. N.. (2024). Seaweed extracts: Enhancing plant resilience to biotic and abiotic stresses. Front. Mar. Sci. 11, 1457500. doi: 10.3389/fmars.2024.1457500

[B61] Kumar K. Debnath P. Singh S. Kumar N. (2023). An overview of plant phenolics and their involvement in abiotic stress tolerance. Stresses 3, 570–585. doi: 10.3390/stresses3030040

[B62] Lal N. Sahu N. (2017). Management strategies of sun burn in fruit crops—A Review. Int. J. Curr. Microbiol. Appl. Sci. 6, 1126–1138. doi: 10.20546/ijcmas.2017.606.131

[B63] Lokanadhan S. Muthukrishnan P. Jeyaraman S. (2012). Neem products and their agricultural applications. J. Biopesticides 5, 72. doi: 10.57182/jbiopestic.5.0.72-76

[B64] Maheshwari D. K. Dheeman S. Agarwal M. (2015). “Phytohormone-producing PGPR for sustainable agriculture,” in Bacterial metabolites in sustainable agroecosystem, ed. Maheshwari D. K.. (Cham, Switzerland: Springer International Publishing), 159–182.

[B65] Mahmood A. Hu Y. Tanny J. Asante E. A. (2018). Effects of shading and insect-proof screens on crop microclimate and production: A review of recent advances. Scientia Hortic. 241, 241–251. doi: 10.1016/j.scienta.2018.06.078

[B66] Mahmoudian M. Rahemi M. Karimi S. Yazdani N. Tajdini Z. Sarikhani S.. (2021). Role of kaolin on drought tolerance and nut quality of Persian walnut. J. Saudi Soc. Agric. Sci. 20, 409–416. doi: 10.1016/j.jssas.2021.04.006

[B67] Manja K. Aoun M. (2019). The use of nets for tree fruit crops and their impact on the production: A review. Scientia Hortic. 246, 110–122. doi: 10.1016/j.scienta.2018.11.018

[B68] Melgarejo-Sánchez P. Martínez J. J. Legua P. Martínez R. Hernández F. Melgarejo P. (2015). Quality, antioxidant activity and total phenols of six Spanish pomegranates clones. Scientia Hortic. 182, 65–72. doi: 10.1016/j.scienta.2014.11.022

[B69] Mishra G. Dash S. P. Mahapatra S. K. Swain D. Rout G. R. (2025). Deeper insights into the physiological and metabolic functions of the pigments in plants and their applications: Beyond natural colorants. Physiologia Plantarum 177, e70168. doi: 10.1111/ppl.70168, PMID: 40159765

[B70] Mitra S. Pathak P. K. (2024). “Origin, production and history,” in Dragon fruit: Botany, production and uses, eds. Mitra S. Pathak P. K.. (Boston, MA: CABI), 7–15.

[B71] Mizrahi Y. (2014). Vine-cacti pitayas: The new crops of the world. Rev. Bras. Fruticultura 36, 124–138. doi: 10.1590/0100-2945-445/13

[B72] Mizrahi Y. Nerd A. Nobel P. S. (1997). Cacti as crops. Hortic. Rev. 18, 291–320.

[B73] Mohamed A. A. Ahmed H. A. Mansour A. H. Omar N. M. (2024). Influence of spraying kaolin and potassium silicate on quality, storability and fungal diseases control of “King Roby” grapevine. Egyptian J. Horticulture 51, 233–250. doi: 10.21608/ejoh.2024.261585.1268

[B74] Mooshammer M. Hofhansl F. Frank A. H. Wanek W. Hämmerle I. Leitner S.. (2017). Decoupling of microbial carbon, nitrogen, and phosphorus cycling in response to extreme temperature events. Sci. Adv. 3, e1602781. doi: 10.1126/sciadv.1602781, PMID: 28508070 PMC5415334

[B75] Munné-Bosch S. Vincent C. (2019). Physiological mechanisms underlying fruit sunburn. Crit. Rev. Plant Sci. 38, 140–157. doi: 10.1080/07352689.2019.1613320

[B76] Naz H. Akram N. A. Ashraf M. Hefft D. I. Jan B. L. (2022). Leaf extract of neem (*Azadirachta indica*) alleviates adverse effects of drought in quinoa (*Chenopodium quinoa* Willd.) plants through alterations in biochemical attributes and antioxidants. Saudi J. Biol. Sci. 29, 1367–1374. doi: 10.1016/j.sjbs.2021.11.054, PMID: 35280556 PMC8913546

[B77] Nerd A. Sitrit Y. Kaushik R. A. Mizrahi Y. (2002). High summer temperatures inhibit flowering in vine pitaya crops (*Hylocereus* spp.). Scientia Hortic. 96, 343–350. doi: 10.1016/S0304-4238(02)00105-2

[B78] Nishikito D. F. Borges A. C. A. Laurindo L. F. Otoboni A. M. B. Direito R. Goulart R. D. A.. (2023). Anti-inflammatory, antioxidant, and other health effects of dragon fruit and potential delivery systems for its bioactive compounds. Pharmaceutics 15, 159. doi: 10.3390/pharmaceutics15010159, PMID: 36678789 PMC9861186

[B79] Nobel P. S. (2003). Environmental biology of agaves and cacti. (Cambridge, UK, Cambridge University Press).

[B80] Nobel P. S. De la Barrera E. (2002). High temperatures and net CO2 uptake, growth, and stem damage for the hemiepiphytic cactus *Hylocereus undatus* . Biotropica 34, 225–231. doi: 10.1111/j.1744-7429.2002.tb00534.x

[B81] Nuzhyna N. Baglay K. Golubenko A. Lushchak O. (2018). Anatomically distinct representatives of Cactaceae Juss. family have different response to acute heat shock stress. Flora 242, 137–145. doi: 10.1016/j.flora.2018.03.012

[B82] Patil A. Kakade V. D. Kalalbandi B. M. Morade A. S. Chavan S. B. Salunkhe V. N. (2024). Mitigating heat stress in dragon fruit in semi-arid climates: The strategic role of shade nets in enhancing fruit yield and quality. Environment Dev. Sustainability, 1–37. doi: 10.1007/s10668-024-04476-x

[B83] Piper C. S. (1944). Soil and plant analysis: A laboratory manual of methods for the examination of soils and the determination of the inorganic constituents of plants (Rev. ed.). (New York, NY: Interscience Publishers).

[B84] Poorter L. Lianes E. Moreno-de Las Heras M. Zavala M. A. (2012). Architecture of Iberian canopy tree species in relation to wood density, shade tolerance and climate. Plant Ecol. 213, 707–722. doi: 10.1007/s11258-012-0038-0

[B85] Przybyłko S. Marszał J. Kowalczyk W. Szpadzik E. (2025). Effect of kaolin clay on post-bloom thinning efficacy, cropping, and fruit quality in ‘Gala vill’ Apple (*Malus × domestica*) cultivation. Agriculture 15, 440. doi: 10.3390/agriculture15040440

[B86] Racsko J. Schrader L. E. (2012). Sunburn of apple fruit: Historical background, recent advances and future perspectives. Crit. Rev. Plant Sci. 31, 455–504. doi: 10.1080/07352689.2012.696453

[B87] Raveh E. Nerd A. Mizrahi Y. (1998). Responses of two hemiepiphytic fruit crop cacti to different degrees of shade. Scientia Horticulturae 73, 151–164. doi: 10.1016/S0304-4238(97)00134-9

[B88] Reig G. Donahue D. J. Jentsch P. (2020). The efficacy of four sunburn mitigation strategies and their effects on yield, fruit quality, and economic performance of ‘Honeycrisp’ Cv apples under Eastern New York (USA) climatic conditions. Int. J. Fruit Sci. 20, 541–561. doi: 10.1080/15538362.2019.1605558

[B89] Reza A. Ahamed T. Miah M. M. U. Ahiduzzaman M. A. (2022). Growth and yield of dragon fruit in Aonla based multistoried fruit production model. Eur. J. Agric. Food Sci. 4, 134–141. doi: 10.24018/ejfood.2022.4.5.565

[B90] Rotondi A. Ganino T. Calderoni A. Rodolfi M. Dhenge R. Morrone L. (2025). Olive Plant Treated with Different Geo-Material Foliar Film (Zeolite and Kaolin Based): Leaf Characteristics and Oil Quality. Horticulturae 11, 338. doi: 10.3390/horticulturae11030338

[B91] Rouphael Y. Colla G. (2018). Synergistic biostimulatory action: Designing the next generation of plant biostimulants for sustainable agriculture. Front. Plant Sci. 9. doi: 10.3389/fpls.2018.01655, PMID: 30483300 PMC6243119

[B92] Santos V. P. D. Coelho Filho M. A. (2024). Effects of sunscreen protection and water management on the physiology and production of ‘Pera’ sweet orange orchards in sub-humid climate. Rev. Bras. Fruticultura 46, e–612. doi: 10.1590/0100-29452024612

[B93] Santoyo G. Guzmán-Guzmán P. Parra-Cota F. I. Santos-Villalobos S. D. L. Orozco-Mosqueda M. D. C. Glick B. R. (2021). Plant growth stimulation by microbial consortia. Agronomy 11, 219. doi: 10.3390/agronomy11020219

[B94] Sardans J. Peñuelas J. (2021). Potassium control of plant functions: Ecological and agricultural implications. Plants 10, 419. doi: 10.3390/plants10020419, PMID: 33672415 PMC7927068

[B95] Sarmin R. A. Khan S. A. K. U. Fatema K. Sultana S. (2018). Effect of neem leaf and banana pulp extracts on shelf life and quality of mango (*Mangifera indica* L.): Effect of plant extract on shelf life of mango. J. Bangladesh Agric. Univ. 16, 343–350. doi: 10.3329/jbau.v16i3.39488

[B96] Selvakumar G. Reetha S. Thamizhiniyan P. (2012). Response of biofertilizers on growth, yield attributes and associated protein profiling changes of blackgram (*Vigna mungo* L. Hepper). World Appl. Sci. J. 16, 1368–1374.

[B97] Shakya R. (2023). “Markers of oxidative stress in plants,” in Ecophysiology of tropical plants, eds. Shakya R.. (Boca Raton, FL: CRC Press), 298–310.

[B98] Shu Z. Shao L. Huang H. Y. Zeng X. Q. Lin Z. F. Chen G. Y.. (2009). Comparison of thermostability of PSII between the chromatic and green leaf cultivars of *Amaranthus tricolor* L. Photosynthetica 47, 548–558. doi: 10.1007/s11099-009-0080-x

[B99] Singleton V. L. Orthofer R. Lamuela-Raventós R. M. (1999). Analysis of total phenols and other oxidation substrates and antioxidants by means of Folin-Ciocalteu reagent. Methods Enzymol. 299, 152–178. doi: 10.1016/S0076-6879(99)99017-1

[B100] Soengas P. Rodríguez V. M. Velasco P. Cartea M. E. (2018). Effect of temperature stress on antioxidant defenses in *Brassica oleracea* . ACS Omega 3, 5237–5243. doi: 10.1021/acsomega.8b00242, PMID: 30023910 PMC6044755

[B101] Sofo A. Dichio B. Montanaro G. Xiloyannis C. (2009). Shade effect on photosynthesis and photoinhibition in olive during drought and rewatering. Agric. Water Manage. 96, 1201–1206. doi: 10.1016/j.agwat.2009.03.011

[B102] Solovchenko A. E. Merzlyak M. N. Pogosyan S. I. (2010). Light-induced decrease of reflectance provides an insight in the photoprotective mechanisms of ripening apple fruit. Plant Sci. 178, 281–288. doi: 10.1016/j.plantsci.2010.01.015

[B103] Subapriya R. Nagini S. (2005). Medicinal properties of neem leaves: A review. Curr. Medicinal Chemistry-Anti-Cancer Agents 5, 149–156. doi: 10.2174/1568011053174828, PMID: 15777222

[B104] Teker T. (2023). A study of kaolin effects on grapevine physiology and its ability to protect grape clusters from sunburn damage. Scientia Hortic. 311, 111824. doi: 10.1016/j.scienta.2022.111824

[B105] Verma V. Ravindran P. Kumar P. P. (2016). Plant hormone-mediated regulation of stress responses. BMC Plant Biol. 16, 86. doi: 10.1186/s12870-016-0771-y, PMID: 27079791 PMC4831116

[B106] Vishwakarma K. Kumar N. Shandilya C. Mohapatra S. Bhayana S. Varma A. (2020). Revisiting plant–microbe interactions and microbial consortia application for enhancing sustainable agriculture: A review. Front. Microbiol. 11. doi: 10.3389/fmicb.2020.560406, PMID: 33408698 PMC7779480

[B107] Wang Y. Cao X. Han Y. Han X. Wang Z. Xue T.. (2022). Kaolin particle film protects grapevine cv. Cabernet Sauvignon against downy mildew by forming particle film at the leaf surface, directly acting on sporangia and inducing the defense of the plant. Front. Plant Sci. 12. doi: 10.3389/fpls.2021.796545, PMID: 35082814 PMC8784833

[B108] Wang W. H. Chen J. Liu T. W. Chen J. Han A. D. Simon M.. (2014). Regulation of the calcium-sensing receptor in both stomatal movement and photosynthetic electron transport is crucial for water use efficiency and drought tolerance in *Arabidopsis* . J. Exp. Bot. 65, 223–234. doi: 10.1093/jxb/ert362, PMID: 24187420 PMC3883291

[B109] White P. J. George T. S. Dupuy L. X. Karley A. J. Valentine T. A. Wiesel L.. (2013). Root traits for infertile soils. Front. Plant Sci. 4. doi: 10.3389/fpls.2013.00193, PMID: 23781228 PMC3678079

[B110] Wylie M. R. Merrell D. S. (2022). The antimicrobial potential of the neem tree *Azadirachta indica* . Front. Pharmacol. 13. doi: 10.3389/fphar.2022.891535, PMID: 35712721 PMC9195866

[B111] Yadav A. Garg S. Kumar S. Alam B. Arunachalam A. (2025). A review on genetic resources, breeding status and strategies of dragon fruit. Genet. Resour. Crop Evol. 72, 2511–2531. doi: 10.1007/s10722-024-02123-y

[B112] Zahedi S. M. Gholami R. Karimi M. Gholami H. (2020). Evaluation of some pomological and biochemical characteristics in Malas Torsh and Yosef-Khani pomegranate cultivars in response to foliar application of kaolin. J. Crops Improvement 22, 487–498. doi: 10.22059/jci.2020.293836.2311

[B113] Zahra N. Hafeez M. B. Ghaffar A. Kausar A. Al Zeidi M. Siddique K. H.. (2023). Plant photosynthesis under heat stress: Effects and management. Environ. Exp. Bot. 206, 105178. doi: 10.1016/j.envexpbot.2022.105178

[B114] Zhang J. Kirkham M. B. (1996). Antioxidant responses to drought in sunflower and sorghum seedlings. New Phytol. 132, 361–373. doi: 10.1111/j.1469-8137.1996.tb01856.x, PMID: 26763632

[B115] Zhishen J. Mengcheng T. Jianming W. (1999). The determination of flavonoid contents in mulberry and their scavenging effects on superoxide radicals. Food Chem. 64, 555–559. doi: 10.1016/S0308-8146(98)00102-2

