# Supplementary material for: Sunburn mitigation in dragon fruit (Hylocereus spp.): unravelling genotype-specific physiological and biochemical responses
Source: Front Plant Sci. 2025 Sep 11;16:1661147. doi: 10.3389/fpls.2025.1661147 (PMC12461260; doi:10.3389/fpls.2025.1661147)
Supplement: Supplementary file 1 [file DataSheet1.docx]

| **Month** | **Temperature (^o^C)** | | **Relative**  **Humidity (%)** | | **U.S.W.B.Class**  **‘A’ Pan**  **Evaporation**  **(mm)** | **Mean wind speed (km/h)** | **Rainfall**  **(mm)** |
| --- | --- | --- | --- | --- | --- | --- | --- |
|  | **Max.** | **Min.** | **08.30**  **hrs.** | **13.30**  **hrs.** |  |  |  |
| January | 28.7 | 15.9 | 88.8 | 45.6 | 3.9 | 4.2 | 0.2 |
| February | 32.0 | 15.2 | 80.9 | 31.3 | 6.2 | 4.7 | 0.0 |
| March | 34.3 | 18.1 | 68.3 | 29.0 | 8.3 | 4.1 | 0.0 |
| April | 36.1 | 19.3 | 65.6 | 27.6 | 9.4 | 4.7 | 0.0 |
| May | 33.4 | 22.8 | 80.9 | 51.5 | 7.1 | 4.2 | 170.9 |
| June | 30.2 | 21.8 | 87.1 | 65.2 | 5.8 | 5.4 | 106.8 |
| July | 28.3 | 21.7 | 89.1 | 71.0 | 4.5 | 8.0 | 58.2 |
| August | 29.0 | 21.6 | 90.5 | 70.9 | 4.6 | 4.5 | 225.9 |
| September | 29.6 | 20.5 | 88.3 | 63.0 | 5.1 | 5.1 | 17.9 |
| October | 28.6 | 20.8 | 92.6 | 71.9 | 3.6 | 2.0 | 391.0 |
| November | 27.4 | 18.4 | 90.5 | 66.8 | 3.5 | 2.9 | 57.9 |
| December | 26.6 | 17.7 | 92.3 | 69.9 | 2.9 | 2.8 | 48.1 |

Supplementary Table 1: Meteorological Data January– December at IIHR, Bengaluru

Supplementary Figure 1: Morphological characteristics of dragon fruit cultivars used in the study: a) red CHESH-D1 and b) white CHESH-W1. The lower images depict the respective plant structures, showing differences in stem morphology and growth habits between the red- and white-pulp cultivars.


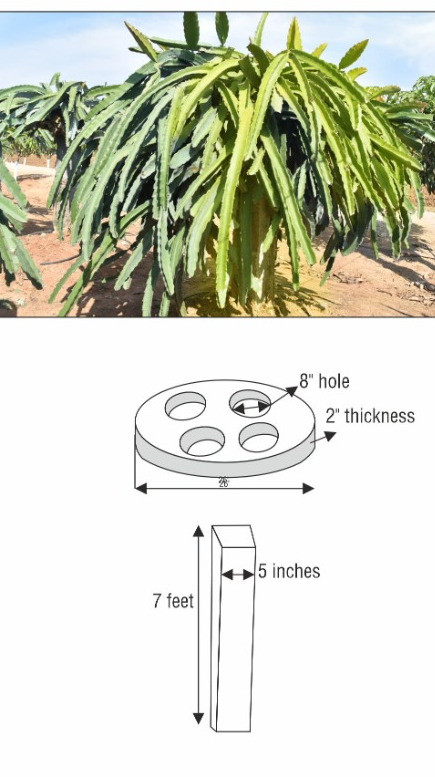


Supplementary Figure 2 Photograph and schematic of the dragon fruit plant trained on a concrete post trellis system. The support structure includes a 7-foot concrete pillar (5-inch width) topped with a circular disc featuring four 8-inch holes to guide plant canopy architecture and promote stability.
